# Response of *Lactobacillus plantarum* WCFS1 to the Gram-Negative Pathogen-Associated Quorum Sensing Molecule *N*-3-Oxododecanoyl Homoserine Lactone

**DOI:** 10.3389/fmicb.2019.00715

**Published:** 2019-04-05

**Authors:** Joseph R. Spangler, Scott N. Dean, Dagmar H. Leary, Scott A. Walper

**Affiliations:** ^1^National Research Council Postdoctoral Fellowships, NRC Research Associateship Programs, Washington, DC, United States; ^2^United States Naval Research Laboratory, Center for Biomolecular Science and Engineering, Washington, DC, United States

**Keywords:** *Lactobacillus plantarum*, quorum sense, *Pseudomonas aeruginosa*, transcriptomics, proteomics, homoserine lactone

## Abstract

The bacterial quorum sensing phenomenon has been well studied since its discovery and has traditionally been considered to include signaling pathways recognized exclusively within either Gram-positive or Gram-negative bacteria. These groups of bacteria synthesize structurally distinct signaling molecules to mediate quorum sensing, where Gram-positive bacteria traditionally utilize small autoinducing peptides (AIPs) and Gram-negatives use small molecules such as acyl-homoserine lactones (AHLs). The structural differences between the types of signaling molecules have historically implied a lack of cross-talk among Gram-positive and Gram-negative quorum sensing systems. Recent investigations, however, have demonstrated the ability for AIPs and AHLs to be produced by non-canonical organisms, implying quorum sensing systems may be more universally recognized than previously hypothesized. With that in mind, our interests were piqued by the organisms *Lactobacillus plantarum*, a Gram-positive commensal probiotic known to participate in AIP-mediated quorum sensing, and *Pseudomonas aeruginosa*, a characterized Gram-negative pathogen whose virulence is in part controlled by AHL-mediated quorum sensing. Both health-related organisms are known to inhabit the human gut in various instances, both are characterized to elicit distinct effects on host immunity, and some studies hint at the putative ability of *L. plantarum* to degrade AHLs produced by *P. aeruginosa.* We therefore wanted to determine if *L. plantarum* cultures would respond to the addition of *N-*(3-oxododecanoyl)-L-homoserine lactone (3OC_12_) from *P. aeruginosa* by analyzing changes on both the transcriptome and proteome over time. Based on the observed upregulation of various two-component systems, response regulators, and native quorum sensing related genes, the resulting data provide evidence of an AHL recognition and response by *L. plantarum*.

## Introduction

Bacteria have been understood to possess a basic level of cell to cell communication for decades (Miller and Bassler, 2001; Waters and Bassler, 2005). The communication phenomenon known as quorum sensing allows organisms to coordinate growth and gene expression efforts, for example, toward a common goal to survive an increasingly harsh environment (Reading and Sperandio, 2006). The traditional model for quorum sensing has been described as the *luxR* system in multiple *Vibrio* spp. (Gray et al., 1994; Bassler et al., 1997; Zhu et al., 2002; Hammer and Bassler, 2003; Waters and Bassler, 2006), where a signaling molecule such as an *N*-acyl homoserine lactone (AHL) is produced and exported into the microenvironment at some undetected basal level. The signaling molecule accumulates over time with proliferation of the producing species until a threshold is reached, whereupon the signal is detected and organisms respond with targeted gene expression. Quorum sensing systems following this model are commonly seen in Gram-negative bacteria such as the aforementioned *Vibrio* spp. to coordinate luminescent protein production or in pathogens such as *Pseudomonas aeruginosa* to coordinate virulence and survival (Gray et al., 1994; Storey et al., 1998; Rumbaugh et al., 1999a,b; Winzer et al., 2000; Rampioni et al., 2006, 2007, 2009; Gao et al., 2017; Kariminik et al., 2017). The downstream genetic response to the signal varies among different organisms, but the general model of signal amplification remains consistent and depends on (1) the synthesis and export of the signal molecule into the environment, (2) environmental accumulation of the signal molecule, (3) diffusion of the signal molecule into neighboring cells, and (4) the interaction of the signal molecule with specific transcription factors resulting in the activation of gene expression. Synthesis of the signal molecule is generally upregulated by transcription factor activation as part of a positive feedback loop, and the number of promoters subsequently induced varies widely among organisms and the specific quorum sensing system involved such that induction rarely results in the upregulation of a single gene (Brint and Ohman, 1995; Medina et al., 2003a,b; Jensen et al., 2006).

Gram-positive organisms are capable of quorum sensing by a different mechanism (Miller and Bassler, 2001). Rather than using AHLs with varying acyl chain lengths as signal molecules, Gram-positive organisms employ the use of short peptides known as autoinducer peptides (AIPs), some of which contain unconventional bonding between specific amino acids to produce unique structures. Considering the multicharge potential of peptides, specific proteins are devoted to the export of autoinducers into the microenvironment. These peptides do not typically diffuse through the membranes like their lactone counterparts given their charged characteristics and the peptidoglycan layer of Gram-positive organisms, thus signal recognition occurs by the activation of a two-component system (Kleerebezem et al., 1997; Sturme et al., 2005; Sturme et al., 2007). The *agr* system in *Staphylococcus aureus* is a well-studied example of peptide-mediated quorum sensing in Gram-positive organisms (Peng et al., 1988; Janzon and Arvidson, 1990; Booth et al., 1995; Papakyriacou et al., 2000; Jensen et al., 2008; Reyes et al., 2011; Marchand and Collins, 2013, 2016), and homologs have been identified in various Lactic Acid Bacteria such as *Lactobacillus sakei*, *Lactobacillus acidophilus*, and *Lactobacillus plantarum* (Kanehisa and Goto, 2000; Sturme et al., 2005, 2007; Fujii et al., 2008). The Lactic Acid Bacteria are known to produce antimicrobial peptides as one response to specific AIP activity as a way to coordinate a defense or fitness mechanism (Diep et al., 1994; Rekhif et al., 1995; Anderssen et al., 1998; Atrih et al., 2001; Maldonado et al., 2002, 2004a,b), while AIP activity in *S. aureus* is reported to contribute to virulence (Booth et al., 1995; Papakyriacou et al., 2000) similar to *P. aeruginosa*. Similar to AHL-mediated sensing, the baseline of genes affected by AIP is unknown given the diversity of organisms that employ this mechanism, and the induction of a single target gene is likely rare.

Quorum sensing traditionally has been split into two classes consisting of AHL- and peptide-mediated signaling assigned to Gram-negative and Gram-positive bacteria, respectively (Miller and Bassler, 2001; Reading and Sperandio, 2006). However, both Gram-negative and Gram-positive bacteria participate in two-component system quorum sensing using autoinducer-2 (AI-2) class molecules, which are structurally unrelated to AHLs and peptides (Reading and Sperandio, 2006). Autoinducer-2 molecules are furanose derivatives of the coenzyme *S-*adenosyl methionine (SAM) whose final active structures vary expectedly among organisms, with some examples containing the element boron (Schauder et al., 2001; Taga et al., 2001; Semmelhack et al., 2004; Vendeville et al., 2005; Pereira et al., 2013). The synthesis of AI-2 involves the *luxS* gene detected in a variety of diverse organisms, leading to the theory that AI-2 molecules represent a universal language for bacterial interspecies communication (Vendeville et al., 2005). The emergence and prevalence of the AI-2 systems seemed to offer a more complete picture of the concept of bacterial communication, where the major bacterial groups of Gram-positive and Gram-negative had their own quorum sensing systems, and a third system existed to facilitate interspecies communication. The existence of such an AI-2 system potentially circumvented the curiosity of whether Gram-positive or Gram-negative organisms could respond to each other’s exclusive signaling molecules. Examples that challenge this presumed exclusivity of AHL and AIP quorum sensing, however, have been identified in the last decade, where AHL production has been noted in Cyanobacteria (Sharif et al., 2008), Archaea (Zhang et al., 2012), and a marine Gram-positive organism from the genus *Exiguobacterium* (Biswa and Doble, 2013). Such discoveries suggest a more universal recognition and utilization of AHLs within the microbial world than previously assumed.

Indeed, AHL recognition by higher order species has been previously observed (Smith et al., 2002a,b; Mathesius et al., 2003; Ritchie et al., 2003, 2005, 2007; Bauer and Mathesius, 2004; Chun et al., 2004; Wagner et al., 2007; Jahoor et al., 2008; Teplitski et al., 2011; Kariminik et al., 2017) with the rationale that the effects imparted by AHLs are due to their structural and functional resemblance to hormones and phytohormones (Teplitski et al., 2011). Within the bacterial kingdom, investigators have previously noted cross-species effects of a *Yersinia enterocolitica* AHL on enterohemorrhagic *Escherichia coli* O157:H7 (Nguyen et al., 2013) and *S. aureus*-derived peptides on both *Enterococcus* spp. (Firth et al., 1994) and *Lactobacillus reuteri* (Lubkowicz et al., 2018). While such responses were mostly unexpected and inexplicable, the latter example could be attributed to the close relation of the *Lactobacillus* and *Staphylococcus* organisms that diverge at the Class level and the apparent endogenous recognition of the *S. aureus* AIP-I despite a lack of annotation for *agr* homologs in *L. reuteri* (Kanehisa and Goto, 2000). Previous attempts to engineer the *agr* system into Firmicutes such as *Bacillus megaterium* were successful and proved the system to be unique to the host (Marchand and Collins, 2013), but the response of *L. reuteri* to the *S. aureus* AIP-I in contrast resulted in repression of an exogenous *agr* promoter rather than stimulation, implying cognate parts of the *agr*-like signaling system may exist in the *Lactobacillus* species used for different purposes than in *S. aureus*. This unexpected result exemplifies the complexity of interspecies quorum sense recognition despite the established genomic characterization of the involved species (Kanehisa and Goto, 2000).

The effects of specific AHLs are presumably localized to cognate and closely related species, at least in terms of an optimized and targeted response based on the number of studies probing the activity of heterologous *luxR* and *lasR* systems in *E.coli* without notable off-target responses (Seed et al., 1995; Latifi et al., 1996; Collins et al., 2005, 2006; Goodson et al., 2015). However, evidence exists of the potential cross-talk, albeit weak, between different AHL-mediated systems based on activity observed in cell-free experiments (Wen et al., 2017; Halleran and Murray, 2018). Considering the above points, it is hard to say universally whether bacteria can sense and respond to any present signaling cues in the microenvironment. In terms of signal fidelity, it makes sense that bacterial species would evolve a unique and uninterceptable signaling regime to coordinate the survival and fitness of itself over others in the community, especially in the context of virulence coordination. *P. aeruginosa* has been well noted to utilize its *lasR*-dependent quorum sensing system to establish biofilm formation and anti-host immunity measures as well as cyanide production to solidify colonization in the face of both host responses and microbial competition (Storey et al., 1998; Pessi and Haas, 2000; Winzer and Williams, 2001). Furthermore, *L. plantarum* has been noted to produce the antimicrobial peptide class of Plantaricins as a result of AIP signaling to similarly diminish bacterial populations in its proximity (Anderssen et al., 1998; Atrih et al., 2001), but this seems to be more of an altruistic endeavor in order to cull the prevalence of organisms potentially harmful to the host (Maldonado et al., 2004b). While the driving force behind the *P. aeruginosa* signaling system may be attributed to cell density given its occurrence in pure cultures (Valdez et al., 2005), the initiation of the aforementioned response by *L. plantarum* is unknown, and could be due to either cell density or accumulation of some unrelated environmental cue (Maldonado et al., 2003, 2004a,b).

We therefore set out to investigate the potential for quorum sensing cross-talk between the two organisms *P. aeruginosa* and *L. plantarum* considering their activity as quorum sensing bacteria, their contrasting roles in the human gut (Matsumoto et al., 1997; Xia et al., 2011), and the effects they elicit on the host immune system (Schultz et al., 2002; Ko et al., 2007; Puertollano et al., 2008; Peral et al., 2010). Furthermore, there has been nominal evidence that *L. plantarum* is putatively capable of degrading AHLs produced by *P. aeruginosa* (Valdez et al., 2005; Peral et al., 2009; Ramos et al., 2012; Ramos et al., 2015), suggesting there could be some mechanism for AHL recognition by *L. plantarum*. The use of deep analytical techniques involving transcriptomics and proteomics allowed us to gather a detailed global picture of the response of *L. plantarum* to the presence of the *P. aeruginosa* AHL *N*-3-oxo-dodecanoyl homoserine lactone (3OC_12_). The wealth of resulting data provided us the opportunity to speculate on both the intracellular ripple effect and timeline for the interspecies response to a predominantly Gram-negative signal by a Gram-positive commensal.

## Materials and Methods

### Reagents

All reagents were obtained from Sigma unless specified otherwise. *N*-3-oxododecanoyl homoserine lactone (3OC_12_, Sigma-Aldrich cat# o9139 – manufacturer reported purity of ≤100%) was maintained in 100 mM stocks in molecular biology grade dimethyl sulfoxide (DMSO, purity ≥99.9%, Sigma Aldrich) at -20°C until use.

### Culture Methods

*Lactobacillus plantarum* WCFS1 (BAA-793) was obtained from American Type Culture Collection and maintained in De Man Rogosa Sharp (MRS) media alone or supplemented with 1.5% agar at 37°C in air.

### 3OC_12_ Challenge

All experiments carried out in triplicate. Individual colonies of *L. plantarum* from MRS agar were grown shaking aerobically at 37°C in 5 mL MRS overnight. The following morning, 1 mL overnight cultures were added to 100 mL fresh MRS and grown similarly for approximately 3 h until an OD_600_ of 0.5, when samples were split into two aliquots of 45 mL of either treated samples with the addition of 3OC_12_ to a final concentration of 100 μM, or control samples with the addition of 0.1% DMSO (v/v). Samples continued to grow at 37°C shaking for aliquot removal and processing at +1, +4, and +7 h following treatment. Proteomic samples were archived by removing 0.5 mL culture in quadruplicate, flash freezing decanted cell pellets in liquid nitrogen and storing at -80°C before analysis. Transcriptomic samples were archived by pelleting 1.5 mL culture and resuspending in 300 μL RNA Later (Qiagen) and storing at -80°C before library preparation.

### RNA Preparation and RNAseq

Frozen sample aliquots were thawed at room temperature and pelleted to remove residual RNA Later before treating with 400 μL Lysozyme Solution (1 mg mL^-1^ lysozyme, 40 mM EDTA, pH 8) for 1 h at 37°C. Lysozyme treated cells were pelleted and resuspended in RNAzol RT (Molecular Research Center) following the manufacturer’s protocol for large RNA isolation. Resulting RNA was resuspended in 20 μL pure water and quantified using a NanoDrop2000 instrument (Thermo Fisher Scientific) and calculations based on absorbance at 260 nm. DNase I (Thermo Fisher Scientific) was added to RNA at 0.5 U μg^-1^ for 30 min at 37°C followed by ethanol precipitation. RNA was again resuspended in 20 μL pure water, quantified by NanoDrop and stored at -80°C. Approximately 1 μg of RNA was subjected to ribosomal RNA depletion using the Ribo-Zero Bacteria kit (Illumina) following the manufacturer’s protocol. Samples were ethanol precipitated after rRNA depletion using glycogen as a carrier and resuspended in 10 μL pure water, quantified by NanoDrop and stored -80°C. Approximately 100 ng rRNA-depleted RNA was used for Library preparation with the NEBNext Ultra Directional RNA Library Prep Kit for Illumina (New England Biolabs), AMPure XP beads (Beckman Coulter, Inc.), and NEBNext Multiplex Oligos for Illumina (New England Biolabs) containing Nextera i7 sequences following the manufacturer’s protocol. Resultant libraries were quantified by the Qubit broad-range protocol (Thermo) and visualized by agarose gel before sequencing by an Illumina MiSeq platform (MiSeq Analyzer v2.5.1.3) using the MiSeq Reagent Kit v3 (Illumina) and the Illumina RNA-seq protocol with paired end reads of 60 bp. Libraries were analyzed by two replicate MiSeq runs generating 47.3 million raw filtered reads.

### RNAseq Analysis

Generated MiSeq reads were then analyzed via the RNAseq pipeline described elsewhere (Li et al., 2018). Read quality control was performed where Phred scores of <20 were trimmed using FastQC and Cutadapt as part of Trim Galore (Krueger, 2015). EDGE-pro (Estimated Degree of Gene Expression in PROkaryotes) (Magoc et al., 2013) for paired end reads was used with default settings on the remaining reads for alignment to the *L. plantarum* WCFS1 genome from NCBI^1^ generating RPKM files and count tables. Each count table was read into R where DESeq2 (Love et al., 2014) was used for differential expression analysis and generating associated statistics as a function of treatment with 3OC_12_ and time. The RNA sequence data is available at NCBI GEO accession GSE124050.

### General Data Analysis

In-house R scripts were used for data analysis and visualization of both RNAseq and proteomics data, including the addition of KEGG annotation (Cock et al., 2009), the generation of boxplots and bar graphs using ggplot2 (Wickham, 2016), and UpSet plots using UpSetR (Lex et al., 2014).

### Proteomics Analysis

Snap frozen pellets were resuspended in 200 μL of 10% n-propanol in 50 mM ammonium bicarbonate, vortexed and sonicated. Cells were lysed in microtubes with 100 μL caps (Pressure Biosciences, Inc., Easton, MA, United States). The tubes were then placed into the HUB-440 Baro-cycler (Pressure Biosciences, Inc., Easton, MA, United States) and lysis was performed by 30 s cycles (20 s ON, 10 s OFF) for 60 cycles at 45 kpsi and 25°C. After lysis, total protein amount in all samples was estimated and adjusted to 12 μg prior to digestion by trypsin in barocycler (60 s cycles – 50 s pressure ON, 10 s pressure OFF, 45 kpsi, 50°C). Digests were dried on speed vac and stored at -20°C prior to LC-MS/MS analysis. Samples were reconstituted in 30 μL of 0.1% formic acid in water and 0.2 μg of total protein was analyzed by LC-MS/MS using U3000 LC coupled to Orbitrap Fusion Lumos mass spectrometer (Thermo Scientific, Waltham, MA, United States). Autosampler loaded sample onto trap column (PepMap 100, C18, 300 μm ID × 5 mm, 5 μm, 100A) via loading pump at 5 μL min^-1^ flow rate and 2% solvent B. Analytical pump set to 300 nL min^-1^ was used to elute peptides from the trap onto analytical column (Acclaim PepMap RSLC, 75 μm ID × 150 mm, C18, 2 μm, 100A). A gradient of 2–60% B in 90 min was used for peptide separation. Solvent A was 0.1% formic acid in water and solvent B was 0.1% formic acid in acetonitrile. Mass spectra were acquired on Fusion Lumos Orbitrap equipped with a Nanospray Flex Ion Source in data-dependent acquisition mode with 3 s cycle times. A survey scan range of 400–1,600 Da was acquired on the Orbitrap detector (resolution 120 K). Maximum injection time was 50 ms and AGC target was 400,000. The most intense ions with charges of 2–7 were fragmented using HCD (higher-energy collisional dissociation), and ions were excluded for 30 s from subsequent MS/MS submission. MS/MS detector was IonTrap with 35 ms injection time and AGC target of 10,000. Resulting spectra were extracted, converted into mgf by ProteoWizard software and searched by Mascot (Matrix Science Inc., London, United Kingdom) against a database containing common standards and contaminants, i.e., trypsin, keratin, etc. (190 protein sequences) and database containing all predicted proteins from *L. plantarum* genome (3,063 sequences). Oxidation of methionine and deamidation of glutamine and asparagine were selected as variable modifications, enzyme was set to trypsin and 3 missed cleavages were allowed. Precursor ion tolerance was set to 100 ppm and fragment ion to 1 Da. Protein identifications were further validated by Scaffold (Proteome Software Inc., Portland, OR, United States). Protein identifications were accepted if they could be established at greater than 90.0% probability and contained at least 2 identified peptides. P Protein probabilities were assigned by the Protein Prophetalgorithm (Nesvizhskii et al., 2003). Proteins that contained similar peptides and could not be differentiated based on MS/MS analysis alone were grouped to satisfy the principles of parsimony. Quantitative analysis was done in Scaffold using emPAIs as an input. Only emPAIs satisfying the probability settings were considered for the analysis (lower scoring matches and probabilities <5% were not included). *t*-test, fold change, and other calculations were performed on emPAIs using Scaffold (Searle, 2010). In order to avoid divide-by-zero errors caused the absence of proteins in fold change calculations, we set missing values to 0.3, as previously described (Bible et al., 2015). The proteomics data is available at ProteomeXchange repository with identifier PDXD012232 and 10.6019/PXD012232.

## Results and Discussion

**FIGURE 1:**
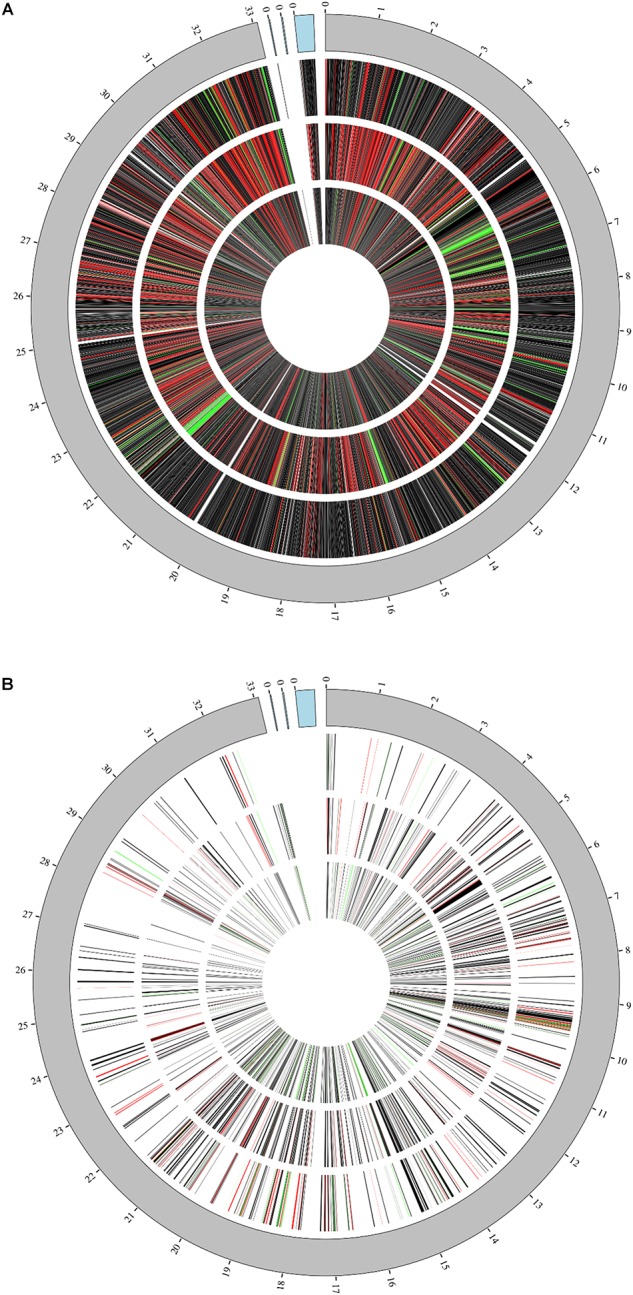
Genome coverage. Coverage of *L. plantarum* genome and its three plasmids (blue region) at 1 h (outermost ring), 4 h (middle ring), and 7 h (innermost ring) by either RNAseq **(A)** or Proteomic analyses **(B)**. Relative fold changes are mapped as red (upregulated), black (unchanged), or green (downregulated).

Cultures of *L. plantarum* WCFS1 were grown from single colonies in overnight cultures of nutrient rich MRS media at 37°C shaking and used the following day to inoculate 100 mL MRS media (1% v/v). The OD_600_ was monitored throughout growth and when it reached 0.5 (approximately 3 h) cultures were split into 45 mL aliquots for treatments. Cultures continued to be incubated at 37°C and aliquots were removed at 1, 4, and 7 h post-treatment for sample preparation. Transcriptomic data was acquired through replicate RNAseq protocols using Illumina MiSeq and resulted in over 47 million raw filtered reads covering the majority (∼93.6% of the annotated 3,174 genes) of the *L. plantarum* WCFS1 genome and its three plasmids (Figure 1A). Proteomic analysis resulting from tandem mass spectrometry on the Orbitrap Fusion Lumos (Thermo) were able to corroborate up to 589 of the identified genes from RNAseq at certain time points yielding coverage illustrated in Figure 1B. The resulting datasets from the two techniques were evaluated both independently and as an integrated response in order to dissect the changes displayed by the organism.

### Transcriptomic Response

**FIGURE 2:**
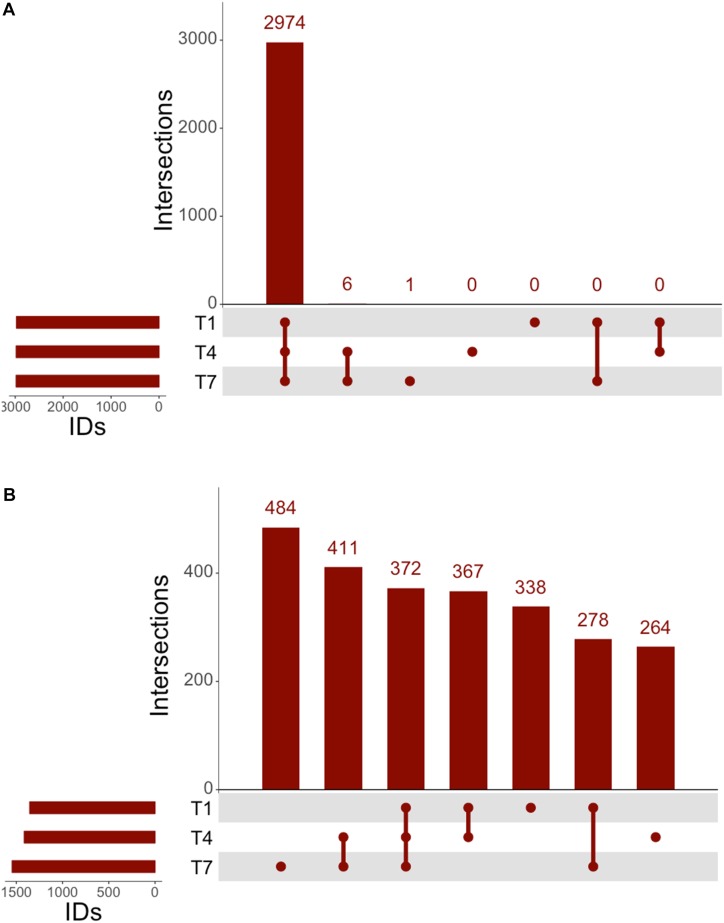
UpSet analysis of RNAseq. **(A)** Genes identified at each timepoint in RNAseq, consisting of 2,974 genes noted at all timepoints. **(B)** Upregulated genes identified by RNAseq consisted of 372 consistently upregulated at all times, with 411 genes upregulated after the 1 h timepoint.

The genomic coverage of RNAseq was relatively consistent throughout all time points (Figure 2A). There were 2,947 genes identified by RNAseq that were common to all three time points, and all genes identified at 1 and 4 h were accounted for at the other times. A large number of the identified genes were determined to be upregulated in comparison to controls (Figure 2B). Of these upregulated genes, 338 were unique to the 1 h samples, 264 were unique to the 4 h samples, and 484 were unique to the 7 h samples, implying that each time point might offer a snapshot of the overall response to 3OC_12_. In addition to genes identified that were unique to specific times, there were also 372 genes observed to be upregulated at all times. It is interesting that there are sets of genes upregulated consistently as well as those unique to specific timepoints, especially considering that our experiments consisted of a single addition of 3OC_12_. Based on the hypothesized longevity of AHLs (Yates et al., 2002; Ramos et al., 2010), such an addition might be characterized as an acute environmental change that would elicit an immediate response that then subsides. These initial results, however, implied that this may have consisted of a cascade of genetic changes over the course of the entire experiment where our sampling times were only able to capture small glimpses of the full response. Additionally, the consistent upregulation of one set of genes might indicate a long-term response relative to what might be expected for purportedly transient stimulus.

**FIGURE 3:**
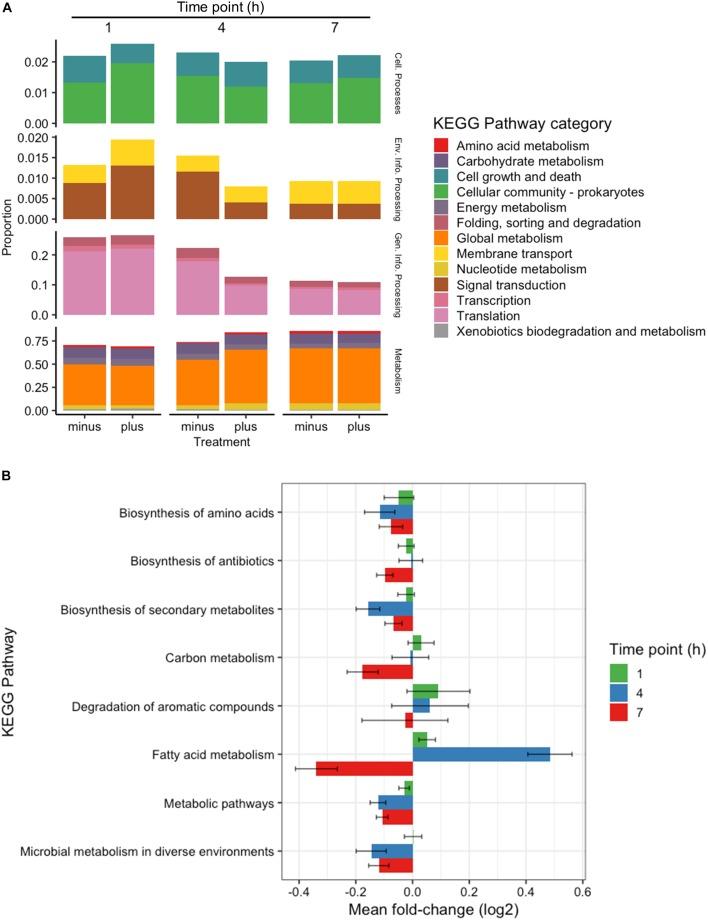
Categorical transcriptomic summary. **(A)** RNAseq hits mapped to different KEGG Pathway Categories at 1, 4, and 7 h. Control samples are marked as “minus” while treated samples are marked as “plus.” **(B)** Changes in gene expression falling within the Global Metabolism category at 1, 4, and 7 h plotted as Mean Log_2_ fold change of 3 biological replicates.

All genes identified throughout the experiments were grouped according to KEGG Pathway Categories (Figure 3A) in order to determine where the majority of the transcriptomic activity was occurring. A large portion of identified genes from all samples fell under the Genetic Information Processing group consisting of Transcription, Translation, Folding, Degradation, Replication and Repair. Nearly a quarter of identified genes were assigned to this category at 1 h, and that abundance decreased as the experiments progressed. Samples analyzed at 1 h after 3OC_12_ addition showed no real changes in Genetic Information Processing, but there was a marked decline in treated samples at 4 h which could indicate the diversion of cellular resources elsewhere for the response. Examples of reductions at 4 h are seen with DNA repair genes such as *exoA*, *recJ*, *tag2*, and *mutL*, protein export genes such as *yidC1*, and RNA degradation genes such as *recQ2* and *rnj*. The activity in General Information Processing at 7 h, however, was the lowest of the whole experiment but equivalent in both treated and control samples indicating a restoration to background levels. The response at 1 h showed a decrease in Cellular Growth genes as cells were initially reacting to the AHL stimulus, but by 4 h these genes had returned to the levels observed in the controls. Increases in Membrane Transport and Signal Transduction categories were also observed at 1 h such as upregulation of the transport associated genes *mtsABC*, *metN*, and *livB* and two-component system genes *rrp11*, *hpk1*, *aad*, *citCEFX*, and *pltKR*. Increases in Cellular Community genes were also observed at 1 h based on the activity of the known quorum sensing genes lp_0783 (oligopeptide transport) and *oroP*. Along the same lines of signaling capabilities, there were increases in the gene *sip1* that is annotated to function in AIP maturation and export. This might be expected from a cell adjusting to a recently changed environment and initiating signal cascades as a response. Both categories of Membrane Transport and Signal Transduction were returned to control levels after 1 h. The most abundant category of genes noted throughout all time points was that of Global Metabolism, accounting for at least half of all identifications. The amount of RNA devoted to Global Metabolism increased at 4 h in treated samples with increases noted for *nagA*, *argCJ*, *pts9AB*, *pgk*, *tpiA*, *enoA*, *galU*, *pgm*, *galE1*, *luxS*, *adk*, and multiple *acc* and *fab* genes for example. The amount of total RNA accounted by this category continued at 7 h, but comparisons with control samples showed it to be of background level abundance.

**FIGURE 4:**
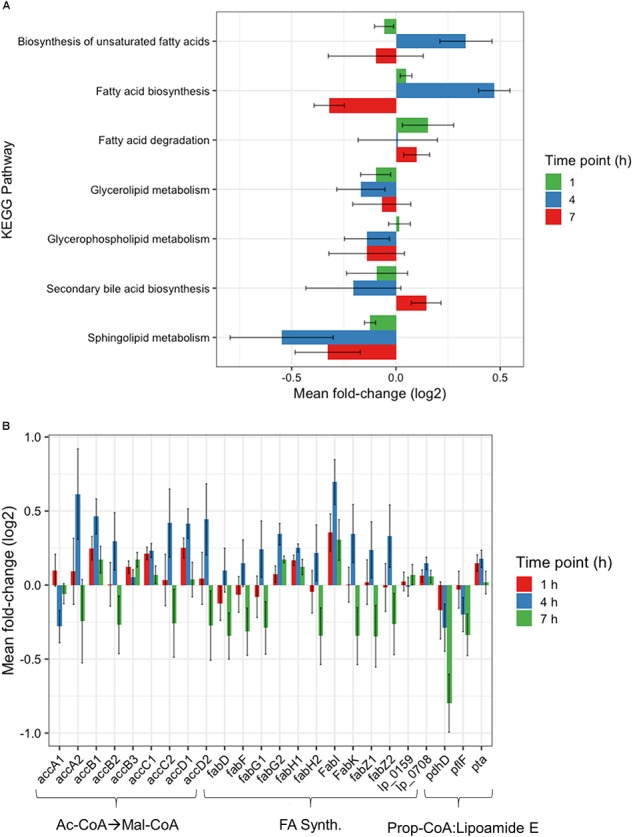
Fatty acid metabolism changes. **(A)** Changes observed by RNAseq within fatty acid metabolism subcategory at 1, 4, and 7 h plotted as Mean Log_2_ fold change of 3 biological replicates. **(B)** RNAseq changes of individual genes involved in Malonyl-CoA synthesis, fatty acid synthesis, and the conversion of propionyl-CoA to Lipoamide E plotted as Mean Log_2_ fold change of 3 biological replicates at 1, 4, and 7 h.

Further investigation of the Global Metabolism response is outlined in Figure 3B. When partitioning the genes of Global Metabolism into 9 further subcategories, it was clear that the increase noted at 4 h was due to activity within Fatty Acid Metabolism. It can be noted here that all subcategories of Global Metabolism revert to a downregulated state at 7 h. Figure 4A shows the further subdivision of Fatty Acid Metabolism into 9 more categories, wherein the Biosynthesis of Unsaturated Fatty Acids and Fatty Acid Biosynthesis groups seem to be the main contributors to the changes noted in Global Metabolism. Both of these groups show upregulation in treated samples at 4 h and downregulation at 7 h. Figure 4B illustrates the components of these two groups and the individual changes noted from treated samples. The incorporated genes make up three groups that represent the conversion from Acetyl-CoA to Malonyl-CoA as a lipid biosynthesis precursor (*acc* genes), the actual synthesis of fatty acids via acyl-carrier protein-containing genes (*fab* genes), and the interconversion of Propionyl-CoA and Lipoamide E (*pdhD*, *pflF*, and *pta*). Each of the genes involved show an increase in activity at 4 h and a decrease at 7 h, with the exception of the Propionyl-CoA involved genes *pdhD* and *pflF*. Previous investigators have noted that environmental stresses can impart changes on membrane hydrophobicity and adhesion capabilities of *Lactobacillus* ssp., indicating that these organisms are prone to alter their fatty acid makeup in response to their environment (Haddaji et al., 2017), therefore this comprehensive activation of Fatty Acid Synthesis genes likely follows this reasoning.

### Proteomic Response

**FIGURE 5:**
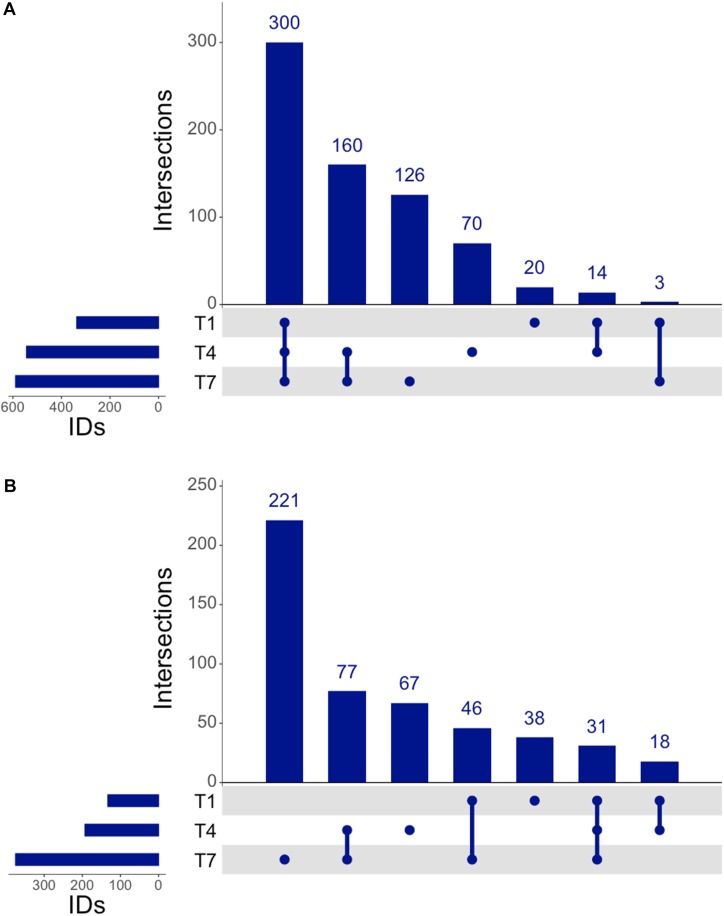
UpSet analysis of proteomics. **(A)** Genes identified at each time by proteomics, consisting of 300 consistently identified genes throughout experiment and an increasing number of identified proteins at each progressive timepoint. **(B)** Upregulated genes identified by proteomics showed 31 consistently upregulated genes throughout the experiment, with 77 genes upregulated after the 1 h timepoint.

The proteomic response of *L. plantarum* WCFS1 cultures to 3OC_12_ was assessed by LC-MS/MS of trypsinized samples on the Orbitrap Fusion Lumos, wherein the output spectra were assigned to known annotated proteins using Mascot (Perkins et al., 1999) and normalized with the Exponentially Modified Protein Abundance Index (emPAI) (Ishihama et al., 2005) to determine a relative abundance of protein per sample similar to strategies used in RNAseq methods. The resulting analysis identified 300 proteins common to samples at all times (Figure 5A). In addition, there were 20 proteins detected unique to the 1 h time point, 70 proteins unique to the 4 h time point, and 126 proteins unique to the 7 h time point. Furthermore, there were 160 proteins that were exclusively identified in the two later time points, and only 3 proteins that were registered at 1 and 7 h only. When comparing treated and control samples, a pattern emerged similar to that seen with RNAseq where each time point contained a unique set of proteins that seemed to increase in number as the experiment progressed (Figure 5B). There were 38 unique upregulated proteins identified at 1 h, 67 at 4 h, and 221 by the 7 h time point. Overall, there were 31 proteins that remained upregulated throughout the experiment. This sweeping trend of increasing translational activity similarly seemed to indicate a 3OC_12_ response.

**FIGURE 6:**
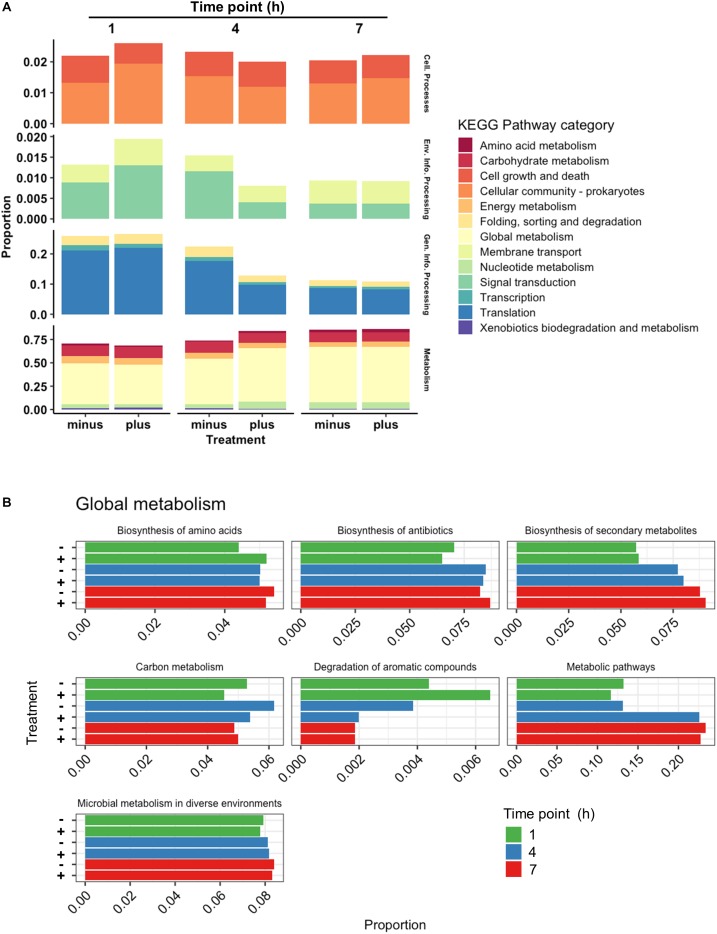
Categorical proteomics summary. **(A)** Identified proteins were mapped to different KEGG Pathway Categories at 1, 4, and 7 h. **(B)** Identified proteins mapped to subcategories within Global Metabolism at 1, 4, and 7 h. Control samples are marked as “minus” while treated samples are marked as “plus.”

Further investigation into the nature of the identified proteins showed an interesting devotion of resources in both control and treated samples (Figure 6A). Treated samples showed a higher abundance of protein at 1 h contributing to Cellular Processes. In particular, genes relating to processes in the Cellular Community category were higher in treated samples including an increased abundance in Lon protease and the oligopeptide transporter lp_0018, but Cell Growth genes were lower (ClpP, ClpX, FtsZ, and FtsA). At 4 h, however, the Cell Growth genes were comparable in both treated and control samples, and Cellular Community genes in treated samples had reduced to below the levels observed in controls. Detected at a lower abundance than the Cellular Process genes were those related to Environmental Information Processing. A clear increase in this category consisting of Signal Transduction and Membrane Transport was observed for treated samples at 1 h based on the abundance of proteins such as DltD, and greatly diminished thereafter. The second highest category of identified proteins was that of Genetic Information Processing. Treated and control samples had devoted roughly an equivalent amount of resources toward this group at 1 h, but at 4 h the translation in treated cells had diminished to nearly half of that observed in controls. This drop could be attributed to reductions in identifications of chaperones DnaK and Hsp3, as well as repair genes XseAB and MutS2, and large ribosomal proteins RplAMNOBU. Considering the values of top upregulated genes identified at all times (Table 1), however, the abundance of some ribosomal proteins (RplKQTVW, RpmABI, RplF, and RpsKNS) and chaperones (Tig) decreased from 1 h to 4 h but remained above the levels of the control samples, indicating a sort of turnover of proteins that are likely involved in the same process over time.

**Table 1:** Upregulated proteins at all timepoints identified by proteomics.

				RNAseq				Proteomics		
Uniprot ID	Locus	ID	Description	1 h	4 h	7 h		1 h	4 h	7 h
EFTU_LACPL	lp_2119	*tuf*	Elongation factor Tu	0.118	0.165	-0.03		0.24	0.01	0.36
F9UM10_LACPL	lp_0789	*gapB*	Glyceraldehyde-3-phosphate dehydrogenase	0.162	0.128	-0.16		0.38	0.42	0.43
F9UMJ6_LACPL	lp_1019	*clpC*	Protease	0.416	0.529	-0.28		0.32	0.06	0.02
F9UNI1_LACPL	lp_1437	*ribA*	3,4-dihydroxy-2-butanone 4-phosphate synthase/GTP cyclohydrolase II	-0.1	-0.16	0.067		0.07	0.03	0.07
F9UP39_LACPL	lp_1675	*fabF*	Lipid metabolism	-0.05	0.371	-0.56		0.21	0.11	1.97
F9UP45_LACPL	lp_1681	*fabI*	lipid metabolism	0.082	1.16	-0.7		0.8	0.57	1.46
F9UPL2_LACPL	lp_1884	*–*	Extracellular protein with LysM peptidoglycan binding domain	0.097	0.043	-0.05		0.19	0.01	0.63
F9UQH9_LACPL	lp_2260	*–*	Extracellular protein	0.039	0.253	-0.05		0.54	0.38	0.7
F9UQT4_LACPL	lp_2393	*–*	Lipoprotein	0.29	0.033	-0.23		0.09	0.06	0.1
F9URR0_LACPL	lp_2794	*–*	Flavodoxin	-0.08	0.264	0.271		0.8	0.27	0.09
F9URU9_LACPL	lp_2847	*–*	Extracellular transglycosylase with LysM peptidoglycan binding domain	0.082	-0.06	-0.13		0.42	0.64	0.05
F9USY1_LACPL	lp_0169	*dhaL*	PEP-glycerone phosphotransferase subunit	-0.33	0.18	0.288		0.56	0.52	1.98
F9UT64_LACPL	lp_0267	*tagD1*	Glycerol-3-phosphate cytidylyltransferase	0.066	0.329	0.125		0.81	0.1	0.02
F9UTB8_LACPL	lp_3214	*–*	Cystathionine ABC transporter	-0.01	-0.11	-0.01		0.3	0.03	0.04
LUXS_LACPL	lp_0774	*luxS*	*S*-ribosylhomocysteine lyase	0.255	0.439	-0.12		0.65	0.3	0.82
PRSA1_LACPL	lp_1452	*prtM1*	Peptidyl-prolyl isomerase	0.168	0.144	0.158		0.44	0.3	0.21
QUEA_LACPL	lp_2285	*queA*	SAM:tRNA ribosyltransferase-isomerase	0.148	0.276	-0.18		0.34	0.02	0.08
RL11_LACPL	lp_0619	*rplK*	50S ribosomal protein L11	0.027	0.675	-0.58		0.34	0.29	1.51
RL17_LACPL	lp_1063	*rplQ*	50S ribosomal protein L17	0.102	0.642	-0.35		0.89	0.31	0.56
RL20_LACPL	lp_1517	*rplT*	50S ribosomal protein L20	0.032	0.537	-0.31		0.7	0.49	0.89
RL22_LACPL	lp_1039	*rplV*	50S ribosomal protein L22	0.026	0.374	-0.64		1.49	0.52	0.66
RL23_LACPL	lp_1035	*rplW*	50S ribosomal protein L23	0.018	0.51	-0.51		0.49	0.16	1.16
RL27_LACPL	lp_1594	*rpmA*	50S ribosomal protein L27	-0	-0.46	-0.23		1.9	1.7	0.19
RL28_LACPL	lp_1624	*rpmB*	50S ribosomal protein L28	-0.18	0.016	-0.24		0.27	0.27	0.15
RL35_LACPL	lp_1516	*rpmI*	50S ribosomal protein L35	0.046	0.44	-0.33		0.22	0.08	0.15
RL6_LACPL	lp_1051	*rplF*	50S ribosomal protein L6	0.079	0.935	-0.58		2.37	0.62	0.31
RS11_LACPL	lp_1061	*rpsK*	30S ribosomal protein S11	0.069	-0.53	-0.48		0.85	0.66	0.8
RS14Z_LACPL	lp_1048	*rpsN*	30S ribosomal protein S14	0.061	0.551	-0.71		1.42	1.5	0.21
RS19_LACPL	lp_1038	*rpsS*	30S ribosomal protein S19	0	-0	-0.47		1.08	0.82	0.08
TIG_LACPL	lp_2118	*tig*	trigger factor, peptidylprolyl isomerase	0.03	0.158	-0.04		1	0.06	0.52
Y1712_LACPL	lp_1712	*xylH*	4-oxalocrotonate tautomerase	-0.23	0.41	0.368		0.91	0.71	0.48


*RNAseq values represent Log_*2*_ fold change of gene abundance. Proteomic values represent Log_*2*_ fold change of emPAI values.*

*Descriptions of the 31 genes identified as upregulated by proteomics at all timepoints along with their corresponding RNAseq Log_*2*_ fold changes*.

The most abundant proteins at all times belonged to the Metabolism category, specifically Global Metabolism, accounting for at least half of the proteins identified similar to what was seen with RNAseq data. While approximately equivalent in abundance at 1 h, proteins identified in this category from treated cells increased notably at 4 h before finally returning to levels similar to controls at 7 h. The activity noted in the Global Metabolism category appeared to come from genes functioning in Metabolic Pathways (Figure 6B), where at 4 h the abundance of proteins in treated samples were near double those identified from controls. Proteins encoded from genes such as *xylH* (putative tautomerase EC 5.3.2.6, lp_1712), *dltC1* (D-alanyl carrier protein 1, lp_2017), *iolE* (inosine dehydratase, lp_3607), *fabI* (enoyl-ACP reductase, lp_1681), and *dak2* (dihydroxyacetone phosphotransferase, lp_0169) are examples of contributors to this increase in abundance. While FabI and Dak2 can both be attributed to different aspects of lipid metabolism, XylH is an enzyme participating in Xylene Degradation, and the lack of other enzymes that *L. plantarum* possesses in this pathway suggests it may be a participant of a community effort to degrade aromatic compounds. The presence of IolE is also curious considering its role in inositol phosphate metabolism, a similarly incomplete pathway within *L. plantarum* itself. DltC1, however, poses an interesting observation given its role in cationic antimicrobial peptide resistance. The expression of *dltC1* is controlled by a two-component system along with other *dlt* genes all found in close proximity to each other on the chromosome, and showed a similar upregulation profile as it’s cohort *dltD*. Indeed the scope of two-component systems is vast and diverse among studied bacteria and have been known to commonly participate in cross-talk (Procaccini et al., 2011), implying an activation of a two-component system by some facet of 3OC_12_ treatment whose downstream cascade may resemble that of cationic antimicrobial peptide resistance.

The top identified proteins throughout the experiment outlined in Table 1 are a more detailed reflection of the summarized categorical changes above. Very few of the proteins identified with the criteria of having an emPAI Log_2_ fold change > 0.25 at one or more time points belong to an Energy Metabolism pathway. The only protein that meets these criteria is GapB, which seemed to steadily increase in abundance throughout the experiment. While there were a number of ribosomal proteins detected at all time points, they do not all maintain similar expression trends based on observed spectra. The most abundant group of proteins identified either belonged to Lipid Metabolism or were involved in cell membrane architecture. Previous investigators have noted *L. plantarum* to undergo changes in membrane composition as a response to environmental stressors (Haddaji et al., 2017), which was interesting based on the presence of LuxS and ClpC in this list of identified proteins. While the latter is a characterized member of the CtsR regulated stress response pathway (Fiocco et al., 2010), the former is a member of various pathways of interest. The LuxS enzyme is a well-characterized *S*-ribosylhomocysteine lyase that plays a part in various amino acid syntheses, but is also responsible for the synthesis of the bacterial interspecies signaling molecule autoinducer-2 (AI-2). Recently LuxS has been linked with the acid stress response in certain strains of *L. plantarum* as well as the increased adherence capabilities of cells (Jia et al., 2018) which has been characterized as a basic bacterial survival mechanism (Hunt et al., 2004). It is intriguing, however, to consider the potential that *L. plantarum* is initiating a quorum sensing event in response to sensing the pathogen-associated 3OC_12_. Based on recent studies, it is not farfetched to deduce that if LuxS is used in the face of environmental stress to increase the fitness of *L. plantarum*, and 3OC_12_ is causing the upregulation of *luxS* in our cultures, then *L. plantarum* possesses some way of recognizing the AHL as an indication of environmental stress.

### Integrated Transcriptomic and Proteomic Response

**FIGURE 7:**
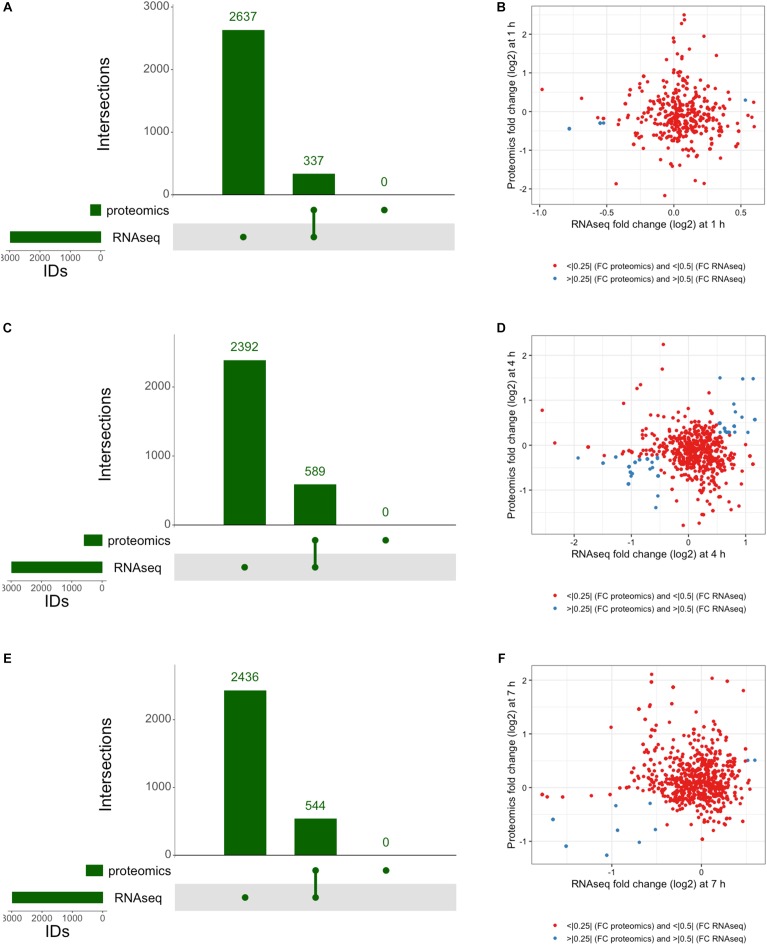
Integrated response analysis. UpSet plot of genes identified by both RNAseq and proteomics at **(A)** 1 h showing 337 genes in common, **(C)** 4 h with 544 genes in common, and **(E)** 7 h with 589 genes in common. Correlation plots of genes identified by RNAseq and Proteomics using threshold cutoffs for genes with Log_2_ fold change > 0.25 for proteomics and > 0.5 for RNAseq (blue dots) at **(B)** 1 h, **(D)** 4 h, and **(F)** 7 h.

**FIGURE 8:**
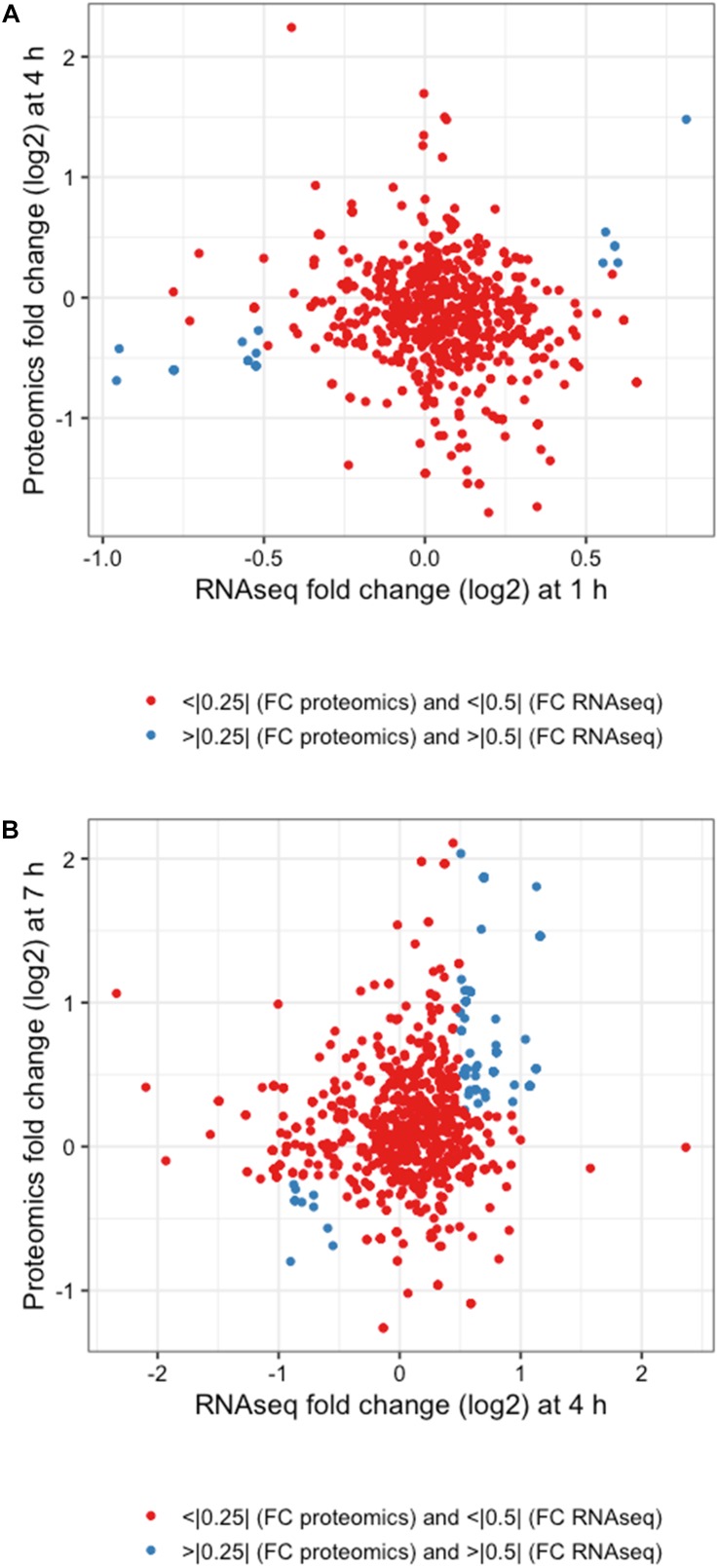
Integrated response analysis with time offset. Correlation plots for **(A)** 1 h RNAseq with 4 h proteomics and **(B)** 4 h RNAseq and 7 h proteomics using threshold cutoffs of Log_2_ fold changes of >0.25 for proteomics and >0.5 for RNAseq (blue dots).

The integration of both proteomic and RNAseq datasets resulted in the reinforcement of previous hypotheses generated from independent analyses. The cross-referencing of both datasets allowed the verification by proteomics of 337 genes previously detected by RNAseq at 1 h, 544 at 4 h, and 589 detected at 7 h (Figure 7A,C,E). The threshold values considered in our analyses were Log_2_-fold change > | 0.25| for proteomic data and > | 0.5| for transcriptomic data in order to gain a broader view of the response using both methods. Correlation plots at specific time points show the number of genes satisfying both threshold criteria at given timepoints (Figure 7B,D,F and Table 1), where the biggest overlap of identifications from proteomics and transcriptomics occurs at 4 h (Figure 7D). When analyzing the data produced across all timepoints by both analyses, we noticed a trend of genes that show upregulation via RNAseq that later appear as protein identifications via proteomics in the next timepoint. We therefore decided to additionally check correlation plots of identifications that met our threshold criteria with a 3 h time offset in mind (Figure 8). As a result of analyzing the correlation of changes in RNAseq at 1 h and proteomics at 4 h (Figure 8A), we identified 13 genes satisfying our previously described criteria wherein all RNAseq results (except those for lp_2433 and lp_0091) met the threshold of *p* < 0.05 (Wald test, Table 2). Using a similar approach to analyze identifications by RNAseq at 4 h and proteomics at 7 h (Figure 8B), we noted 45 genes of interest, of which 71% similarly showed *p* < 0.05 (Wald test, Table 2).

**Table 2:** Genes identified by integrated time offset analysis between RNAseq and proteomics.

				RNAseq	Proteomics
Uniprot ID	Locus	ID	Description	1 h	4 h
F9US34_LACPL	lp_0002	*dnaN*	Beta sliding clamp	-0.78	-0.60
F9UL45_LACPL	lp_0575	*pts9AB*	PTS system, mannose-specific EIIAB component (EC 2.7.1.69)	-0.52	-0.57
F9UL47_LACPL	lp_0577	*pts9D*	PTS system, mannose-specific EIID component (EC 2.7.1.69)	-0.55	-0.52
F9UL90_LACPL	lp_0627	–	Prophage P1 protein 4	-0.96	-0.69
F9UL91_LACPL	lp_0628	–	Prophage P1 protein 5, superinfection exclusion (Cell surface N-anchored)	-0.57	-0.37
CH10_LACPL	lp_0727	*groS*	10 kDa chaperonin (GroES protein) (Protein Cpn10)	0.60	0.29
CH60_LACPL	lp_0728	*groL*	60 kDa chaperonin (GroEL protein) (Protein Cpn60)	0.55	0.29
F9UMR1_LACPL	lp_1097	*mtsA*	Manganese/zinc ABC transporter, substrate binding protein	0.56	0.54
F9UMX1_LACPL	lp_1173	–	UDP-N-acetylglucosamine 2-epimerase (EC 5.1.3.14)	0.59	0.43
F9UQX3_LACPL	lp_2433	–	Prophage P2a protein 24 endodeoxyribonuclease RusA-like	-0.95	-0.42
F9US12_LACPL	lp_2925	–	Cell surface protein, LPXTG-motif cell wall anchor	-0.52	-0.46
F9UTB1_LACPL	lp_3204	*nupC*	Pyrimidine nucleoside transport protein	-0.52	-0.27
F9ULM2_LACPL	lp_3679	–	Cell surface protein, CscB family	0.81	1.48
F9USS0_LACPL	lp_0091	–	Pyridoxamine 5^′^-phosphate oxidase, FMN-binding	-0.81	-0.39

				**4 h**	**7 h**

F9USY7_LACPL	lp_0175	*mdxE*	Maltooligosaccharide transport system substrate-binding protein	-0.88	-0.26
MTLD_LACPL	lp_0233	*mtlD*	Mannitol-1-phosphate 5-dehydrogenase	-0.71	-0.34
F9UT36_LACPL	lp_0235	–	Hypothetical protein	-0.55	-0.69
F9UKW1_LACPL	lp_0479	–	Hypothetical protein	0.55	1.09
F9UL19_LACPL	lp_0546	*hprT*	Hypoxanthine phosphoribosyltransferase	0.50	0.93
HSLO_LACPL	lp_0548	*hsp33*	Heat shock protein	-0.86	-0.30
F9UL33_LACPL	lp_0562	*nagA*	*N*-acetylglucosamine -6-phosphate deacetylase	0.70	0.37
RL11_LACPL	lp_0619	*rplK*	Large subunit ribosomal protein L11	0.67	1.51
Y699_LACPL	lp_0699	–	Hypothetical protein	0.54	1.08
F9ULU4_LACPL	lp_0709	*galE1*	UDP-glucose 4-epimerase	0.58	0.39
F9ULY4_LACPL	lp_0757	*galU*	UTP–glucose-1-phosphate uridylyltransferase	0.63	0.40
F9ULZ5_LACPL	lp_0771	–	Metal-dependent phosphohydrolase, HD family	0.54	0.49
CLPP_LACPL	lp_0786	*clpP*	Clp protease	0.58	0.65
PGK_LACPL	lp_0790	*pgk*	Phosphoglycerate kinase	0.55	0.54
TPIS_LACPL	lp_0791	*tpiA*	Triosephosphate isomerase	0.70	1.87
F9UM53_LACPL	lp_0837	–	Hypothetical protein	0.79	0.89
F9UMC6_LACPL	lp_0927	–	Membrane protein	0.54	0.26
SYS2_LACPL	lp_1012	*serS2*	Seryl-tRNA synthetase	0.57	0.36
RL23_LACPL	lp_1035	*rplW*	Large subunit ribosomal protein L23	0.51	1.16
RS8_LACPL	lp_1050	*rpsH*	Small subunit ribosomal protein S8	0.95	0.43
RL6_LACPL	lp_1051	*rplF*	Large subunit ribosomal protein L6	0.93	0.31
RL18_LACPL	lp_1052	*rplR*	Large subunit ribosomal protein L18	1.04	0.75
RS5_LACPL	lp_1053	*rpsE*	Small subunit ribosomal protein S5	0.80	0.70
KAD_LACPL	lp_1058	*adk*	Adenylate kinase	1.13	0.54
RL17_LACPL	lp_1063	*rplQ*	Large subunit ribosomal protein L17	0.64	0.56
F9UN65_LACPL	lp_1280	*araT1*	Aminotransferase	-0.86	-0.38
RL20_LACPL	lp_1517	*rplT*	Large subunit ribosomal protein L20	0.54	0.89
FABZ_LACPL	lp_1670	*fabZ1*	3-hydroxyacyl-ACP dehydratase	0.51	0.81
F9UP40_LACPL	lp_1676	*accB2*	AcCoA carboxylase biotin carboxyl carrier protein	0.59	1.07
F9UP42_LACPL	lp_1678	*accC2*	AcCoA carboxylase, biotin carboxylase subunit	0.78	0.52
ACCD2_LACPL	lp_1679	*accD2*	AcCoA carboxylase carboxyl transferase subunit beta	0.80	0.66
F9UP44_LACPL	lp_1680	*accA2*	AcCoA carboxylase carboxyl transferase subunit alpha	1.07	0.42
F9UP45_LACPL	lp_1681	*fabI*	Enoyl-ACP reductase I	1.16	1.46
MSRB_LACPL	lp_1836	*mrsB*	Peptide-methionine (R)-S-oxide reductase	0.71	0.34
F9UPT6_LACPL	lp_1972	–	Hypothetical protein	0.63	0.49
Y2062_LACPL	lp_2062	–	Hypothetical protein	0.53	0.51
F9UQP6_LACPL	lp_2342	–	Xre family transcriptional regulator	0.62	0.54
F9UQT3_LACPL	lp_2391	–	Hypothetical protein	-0.71	-0.42
F9USG8_LACPL	lp_3045	–	Short-chain dehydrogenase/oxidoreductase, classical SDR family	0.65	0.30
GPMA2_LACPL	lp_3170	*pmg9*	2,3-bisphosphoglycerate-dependent phosphoglycerate mutase	0.55	1.01
F9UTA0_LACPL	lp_3191	*rrp11*	Two component system response regulator	0.51	2.04
F9UTB7_LACPL	lp_3211	–	Cystine transport system ATP-binding protein	-0.59	-0.57
F9UU98_LACPL	lp_3419	–	Hypothetical protein	-0.90	-0.80
F9ULM2_LACPL	lp_3679	–	Cell surface protein precursor, CscB family	1.13	1.81


*RNAseq values represent Log_*2*_ fold change in transcript abundance. Proteomic values represent Log_2_ fold change in emPAI values.*

*Descriptions of genes reaching threshold criteria of Log_*2*_ fold change > 0.25 in proteomic analysis and > 0.5 in RNAseq analysis for both the 1 h RNAseq and 4 h proteomics set and the 4 h RNAseq and 7 h proteomics set*.

The genes identified satisfying Log_2_ fold change thresholds for both RNAseq and proteomics with offset times are summarized in Table 2. Changes in RNAseq that were verified with proteomics consisted of upregulation of membrane-associated proteins encoded by lp_3679 (CscB family cell surface protein) and *mtsA* (metal transporter), chaperones GroES and GroEL, and lp_1173 (UDP-*N*-acetylglucosamine 2-epimerase) that could be involved in membrane architecture. The transcriptional changes at 4 h that were corroborated with a similar protein response at 7 h consisted of a variety of processes. There seemed to be an emphasis on ATP generation and conservation among the observed changes in abundance. Genes such as *mtlD* involved in fructose/mannose metabolism were downregulated, as were genes such as *mdxE* responsible for maltooligosaccharide transport and lp_3211 involved in cystine transport. ABC transporters similar to these have been shown to be downregulated during the onset of stationary phase-related environmental stressors (Cohen et al., 2006) based on the assumption that they require ATP, which at that time is a precious commodity. When checking the rest of our data for this trend of ATP conservation we found that of the 93 ABC transporters detected by RNAseq at 4 h, all but 16 were downregulated compared to controls, and 11 of the 14 detected by proteomics at 7 h were also downregulated. While *L. plantarum* cells remained in log phase of growth throughout these experiments, it’s possible a similar environmental pressure was asserted on the cells causing them to reprioritize their use of ATP. The genes *pgk*, *tpiA* and *pmg9* involved in glycolysis were upregulated, presumably to facilitate an influx of metabolites into this pathway to more readily produce ATP. Also noted was an increase in the abundance of *adk.* This gene encodes the adenylate kinase enzyme responsible for generating ADP and dADP as substrates for *ndk* to convert into ATP. Increases were also noted in the levels of *hprT*, which is responsible for the generation of GMP from guanine as a precursor to GTP synthesis. In addition to energy conservation, genes involved in nucleotide sugar metabolism such as *nagA*, *galE1*, and *galU* were all observed to be upregulated during this portion of the response as well. UDP-glucose 4-epimerase, encoded by *galE1*, is also a participant in the Leloir pathway and has been observed to be upregulated during late log phase of growth as cells alter their membrane structure to deal with the changing environment (Cohen et al., 2006). Like what was observed with the ABC transporters, cells did not enter stationary phase but it is possible they are adapting to environmental stressors by altering their membrane composition (Haddaji et al., 2017). This hypothesis is reinforced by the upregulation of a number of genes involved in the conversion of Acetyl-CoA to Malonyl CoA (*accA2*, *accB2*, *accC2*, and *accD2*) and the subsequent conversion of Malonyl-CoA to different fatty acids (*fabZ1*, *fabI*, and lp_3045).

### Stress Response

Stress responses consist of a variety of differentially expressed genes and can originate from a number of different stimuli as mentioned above. While there have been a number of studies on *Lactobacillus* ssp. responding to various stresses such as oxidative stress (Serrano et al., 2007), general acid stress (Heunis et al., 2014; Seme et al., 2015), phenolic acid stress (Gury et al., 2009), lactic acid stress (Pieterse et al., 2005), alkaline stress (Lee et al., 2011), metal stress (Tong et al., 2017), growth phase transition stress (Cohen et al., 2006), and the general Classes I and III stress responses (Van Bokhorst-van de Veen et al., 2013), most of these studies indicate a large number of participating genes with no apparent timeframe on induction of the response or the restoration to the pre-stimulus state presumably due to the nature of the individual stress tested. Although 3OC_12_ is not traditionally considered a stressor, we observed similarities in gene and protein activity with stress responses characterized in similar organisms when we analyzed our cultures after exposure to this AHL.

One of the top identified genes in both RNAseq and proteomics was that of *mrsB* (Table 2). The enzyme peptide-methionine (R)-S-oxide reductase encoded by this gene is responsible for reducing oxidized methionine caused by reactive oxygen species. Aside from the specific activity of MrsB, thioredoxin is a more general oxidative-stress response protein whose activity has been established in *L. plantarum* (Serrano et al., 2007). While the investigators emphasized *trxB1* to be the key player involved in the stress response, our data showed significant upregulation of the genes *trxB*, *trxA2*, and *trxH* at 1 and 4 h in transcriptomics. The collective upregulations of *trxB*, *trxA2*, *trxH*, and *mrsB* indicate the cells to be responding similarly as they might in oxidative stress conditions.

The universal stress protein (Usp) family involves nucleotide-binding proteins that function in various non-specific stress conditions. Research has been done in particular on their involvement with phenolic acid stress in *L. plantarum* (Gury et al., 2009) focusing on the activity of Usp1. There are 10 uncharacterized universal stress proteins in *L. plantarum* WCFS1, and all but 1 show upregulation to some extent at 1 h by RNAseq. Of those 9, 4 are upregulated still at 4 h while 3 are downregulated, and 5 are upregulated at 7 h while only 1 is downregulated. Without further elucidation of the roles of these individual universal stress proteins, no conclusions can be drawn about the nature of the stress response displayed. The only thing that can be concluded is that it does not follow the pattern observed from stress brought on by the presence of organic acids, as the Usp immediately downstream of the PadR repressor studied (Gury et al., 2009) displayed changes opposite to those that would be expected from such a response.

Studies on the acid stress response in *L. plantarum* strains (Heunis et al., 2014) have shown increasing activity in a number of seemingly unrelated genes, characterizing a profile for the adaptation to the acidic environment that includes changes in energy metabolism, for example. Of the 18 genes previously observed as upregulated in response to acid stress (Heunis et al., 2014), only 3 are upregulated in our RNAseq data at 1 h (*pmi* or lp_2384, *ldhL1* or lp_0537, and *pta* or lp_0807), 2 at 4 h (*nagB* or lp_0226, and *ldhL2* or lp_1101), and 1 at 7 h (*acdH* or lp_0329). The genes upregulated at 1 h and 4 h are involved in carbohydrate metabolism, while *acdH* encodes a putative acetaldehyde dehydrogenase. Furthermore, the *ldhL* gene observed as upregulated at multiple times encodes the enzyme lactate dehydrogenase responsible for the reduction of pyruvate to lactate, which is the hallmark of the Lactic Acid Bacteria as a method of regenerating NAD^+^. Another acid-induced stress marker in *L. plantarum* has been shown to be LuxS (Jia et al., 2018). While it didn’t reach the thresholds set above (Log_2_ fold change > 0.5), RNAseq was able to identify the slight but significant *luxS* upregulation occurring at 1 h (Log_2_ fold change = 0.25, or +19% increase; *p* = 2 × 10^-5^) and at 4 h (Log_2_ fold change = 0.44, or +37% increase; *p* = 0.002). Proteomic analysis was further able to confirm LuxS upregulation throughout all treated samples. While previous studies have shown LuxS to be involved in acid response, we did not observe any other significant markers for such stress. Based on the function of LuxS, however, it is highly probable that other factors could be responsible for its induction.

Studies on transcriptomic activity during growth phase transitions in *L. plantarum* provide a long list of differentially expressed genes (Cohen et al., 2006). While cultures in our experiments never entered stationary phase of growth, there were some similar changes in gene expression observed in our experiments to those noted in late-log phase and stationary phase. Examples include *dacA1*, *hpk11*, and *rrp11* which all were noted to increase at 1 and 4 h in RNAse q. The Rrp11 protein functions as a two-component system response regulator that was also identified by proteomics at 7 h, further reinforcing its upregulation over time. These three genes have been hypothesized to be involved in a two-component system involved in cell wall maintenance, adding to the list of similarly tasked genes that have been previously discussed above. Cohen et al. (2006) also noted the high abundance of Plantaricin genes throughout growth that decreased with the onset of stationary phase. Our RNAseq data showed that of the 21 annotated *pln* genes, all were downregulated at 1 h, 5 were upregulated at 4 h, and by 7 h there were 13 that were upregulated. As all time points in our experiments represent log phase of growth, the increasing expression of Pln genes over time does not disagree with previous observations of their phase-specific expression patterns. Their upregulation in comparison to controls, however, makes the activity of these Plantaricin genes interesting. Considering previous instances when *L. plantarum* has employed the use of Plantaricins such as when grown in co-culture with other bacteria (Maldonado et al., 2003, 2004a,b), the idea of 3OC_12_ stimulating a Plantaricin response is an intriguing example of a stress-induced defense mechanism. Cohen et al. (2006) also noted a small group of stress-related proteins that peak during stationary phase, specifically *dps1*, *grpE*, *clpP*, and *kat*, all of which are upregulated at 1 h in our experiments. With exception of *clpP*, all of these genes are also upregulated at 4 h as well.

General stress responses can be grouped into classes based on their regulators. The causes for such responses are traditionally attributed to heat shock or general stress conditions (Derre et al., 1999). The Class I and III stress response regulons have recently been characterized in *L. plantarum* by a transcriptomic analysis of single- and double-mutants of their respective regulators HrcA and CtsR (Van Bokhorst-van de Veen et al., 2013). The Class I response regulon governed by the HrcA repressor showed the involvement of genes such as the chaperones *groS* and *groL*, the *hsp1* small heat shock protein, and three putative genes annotated as an integrase/recombinase (lp_1268), a CAAX family membrane-bound protease (lp_0726), and an uncharacterized protein (lp_1880). Each of these Class I regulon members with the exception of the protease and the recombinase were observed to be upregulated via RNAseq at 1 and 4 h. Proteomics further confirmed increased numbers of GroES and GroEL at 4 h and increased Hsp1 at 7 h. The Class III response regulon that is controlled by the repressor CtsR has been studied a couple of times in *L. plantarum* (Fiocco et al., 2009; Fiocco et al., 2010; Van Bokhorst-van de Veen et al., 2013), and consists mainly of the Clp proteases and Clp ATPases. The existing *clp* genes in the *L. plantarum* WCFS1 genome (*clpPCEBXL*) were all determined to be upregulated by RNAseq at 1 h (with the exception of *clpL*) and at 4 h (with the exception of *clpE*), and all were downregulated compared to controls at 7 h in our experiments. Proteomic analysis was further able to confirm the upregulation of ClpP at 7 h. Aside from *clp* genes, CtsR has also been shown in *L. plantarum* to control the small heat shock protein encoded by *hsp1*, the protease subunits encoded by *hslU* and *hslV*, a tyrosine recombinase *xerC*, and an annotated aldose-1-epimerase (lp_1843) (Van Bokhorst-van de Veen et al., 2013). The putative aldose 1-epimerase (lp_1843), the two protease subunits (lp_1845 and lp_1846) and the tyrosine recombinase (lp_1847) all fall within the same region of the chromosome and are likely regulated by a single promoter. As such, our RNAseq data show these four genes are upregulated at 1 h, but only the distal lp_1843 remained upregulated by 4 h. Proteomics, however, were only able to confirm the upregulation of the protease subunits HslU and HslV.

Despite the abundance of genes described as part of various stress responses, the responses we observed from our cultures did not perfectly align with any established stress response. Although 3OC_12_ is not traditionally considered a stressor, and the genes upregulated in response to its addition to cultures of *L. plantarum* are do not traditionally follow any single previously defined stress response pathway, we consider this AHL sensing to be a form of a stress response based on our observations of downregulated cellular growth genes in addition to upregulation of previously characterized stress response genes compared to untreated controls. In particular, this AHL response shares similarities with a number of different characterized stress responses, namely oxidative stress responses, general Classes I and III stress responses, and late growth stage transition responses. Based on the nature of our experiments we can conclude that our cultures were free of any traditional initiator of these set responses, and therefore these findings represent a unique response that may borrow facets of other commonly used response pathways to achieve the most desirable survival phenotype.

## Conclusion

Here we have shown that *L. plantarum* WCFS1 is capable of sensing the Gram-negative quorum sensing molecule 3OC_12_ from *P. aeruginosa*. Transcriptomic and proteomic analyses indicate a number genes specifically upregulated as a result of 3OC_12_ titration into pure cultures of *L. plantarum*. The majority of genes identified by both methods fall within the category of Global Metabolism with an emphasis on Fatty Acid Synthesis, although a number of identified genes also hint at the organism’s alteration of energy metabolism in order to conserve ATP similar to what might occur in a transition to stationary phase of growth. Further changes registered by both methods include genes consistent with both a Classes I and III stress response traditionally caused by general environmental stress. While no stress response profiles could be perfectly matched to previous omic investigations of stress responses, our data showed similarities with cellular responses to oxidative stress and those associated with growth phase transitions, among others, such as thioredoxin activity, fatty acid synthesis, and membrane maintenance. Based on the genes identified we can conclude that the cell is responding to environmental stress unlike other previously established responses. The upregulation of the AI-2 synthesizing enzyme LuxS is an intriguing occurrence that implicates the attempt of *L. plantarum* to externalize a cell signaling event as a response to AHL addition. The upregulation of the Plantaricin genes is a similar event that when taken together with the *luxS* activity paints the picture of a probiotic organism initiating a defense mechanism in response to a pathogen-associated small molecule. We have therefore provided evidence that *L. plantarum* responds to the presence of 3OC_12_ by initiating multiple quorum sensing systems of its own given the *luxS* activity and the upregulation of Plantaricin that results from AIP signaling. In addition, the induction of two-component systems and of multiple putative transcription factors further hints at the complex cell signaling cascade initiated by this AHL.

## Author Contributions

JS, SD, DL, and SW contributed to the experimental work described in this manuscript and the preparation of the manuscript. JS and SD cultured bacteria and prepared samples for transcriptomics and proteomics. JS performed library preparation and transcriptomics studies. DL was responsible for all proteomics work. SD performed data compiling and preliminary analysis. SW directed experimental design and analysis.

## Conflict of Interest Statement

The authors declare that the research was conducted in the absence of any commercial or financial relationships that could be construed as a potential conflict of interest.

## Footnotes

1 RefSeq NC_004567.2, NC_006375.1, NC_006376.1, NC_006377.1.

## References

[B1] Anderssen E. L. Diep D. B. Nes I. F. Eijsink V. G. Nissen-Meyer J. (1998). Antagonistic activity of *Lactobacillus plantarum* C11: two new two-peptide bacteriocins, plantaricins EF and JK, and the induction factor plantaricin A. *Appl. Environ. Microbiol.* 64 2269–2272. 9603847 10.1128/aem.64.6.2269-2272.1998 PMC106311

[B2] Atrih A. Rekhif N. Moir A. J. Lebrihi A. Lefebvre G. (2001). Mode of action, purification and amino acid sequence of plantaricin C19, an anti-Listeria bacteriocin produced by *Lactobacillus plantarum* C19. *Int. J. Food Microbiol.* 68 93–104. 10.1016/S0168-1605(01)00482-2 11545225

[B3] Bassler B. L. Greenberg E. P. Stevens A. M. (1997). Cross-species induction of luminescence in the quorum-sensing bacterium Vibrio harveyi. *J. Bacteriol.* 179 4043–4045. 10.1128/jb.179.12.4043-4045.1997 9190823 PMC179216

[B4] Bauer W. D. Mathesius U. (2004). Plant responses to bacterial quorum sensing signals. *Curr. Opin. Plant Biol.* 7 429–433. 10.1016/j.pbi.2004.05.008 15231266

[B5] Bible A. N. Khalsa-Moyers G. K. Mukherjee T. Green C. S. Mishra P. Purcell A. (2015). Metabolic adaptations of *Azospirillum brasilense* to oxygen stress by cell-to-cell clumping and flocculation. *Appl. Environ. Microbiol.* 81 8346–8357. 10.1128/AEM.02782-15 26407887 PMC4644645

[B6] Biswa P. Doble M. (2013). Production of acylated homoserine lactone by gram-positive bacteria isolated from marine water. *FEMS Microbiol. Lett.* 343 34–41. 10.1111/1574-6968.12123 23489290

[B7] Booth M. C. Atkuri R. V. Nanda S. K. Iandolo J. J. Gilmore M. S. (1995). Accessory gene regulator controls Staphylococcus aureus virulence in endophthalmitis. *Invest. Ophthalmol. Vis. Sci.* 36 1828–1836. 7635657

[B8] Brint J. M. Ohman D. E. (1995). Synthesis of multiple exoproducts in *Pseudomonas aeruginosa* is under the control of RhlR-RhlI, another set of regulators in strain PAO1 with homology to the autoinducer-responsive LuxR-LuxI family. *J. Bacteriol.* 177 7155–7163. 10.1128/jb.177.24.7155-7163.1995 8522523 PMC177595

[B9] Chun C. K. Ozer E. A. Welsh M. J. Zabner J. Greenberg E. P. (2004). Inactivation of a *Pseudomonas aeruginosa* quorum-sensing signal by human airway epithelia. *Proc. Natl. Acad. Sci. U.S.A.* 101 3587–3590. 10.1073/pnas.0308750101 14970327 PMC373506

[B10] Cock P. J. Antao T. Chang J. T. Chapman B. A. Cox C. J. Dalke A. (2009). Biopython: freely available Python tools for computational molecular biology and bioinformatics. *Bioinformatics* 25 1422–1423. 10.1093/bioinformatics/btp163 19304878 PMC2682512

[B11] Cohen D. P. Renes J. Bouwman F. G. Zoetendal E. G. Mariman E. De Vos W. M. (2006). Proteomic analysis of log to stationary growth phase *Lactobacillus plantarum* cells and a 2-DE database. *Proteomics* 6 6485–6493. 10.1002/pmic.200600361 17115453

[B12] Collins C. H. Arnold F. H. Leadbetter J. R. (2005). Directed evolution of vibrio fischeri LuxR for increased sensitivity to a broad spectrum of acyl-homoserine lactones. *Mol. Microbiol.* 55 712–723. 10.1111/j.1365-2958.2004.04437.x 15660998

[B13] Collins C. H. Leadbetter J. R. Arnold F. H. (2006). Dual selection enhances the signaling specificity of a variant of the quorum-sensing transcriptional activator LuxR. *Nat. Biotechnol.* 24 708–712. 10.1038/nbt1209 16715074

[B14] Derre I. Rapoport G. Msadek T. (1999). CtsR, a novel regulator of stress and heat shock response, controls clp and molecular chaperone gene expression in gram-positive bacteria. *Mol. Microbiol.* 31 117–131. 10.1046/j.1365-2958.1999.01152.x 9987115

[B15] Diep D. B. Havarstein L. S. Nissen-Meyer J. Nes I. F. (1994). The gene encoding plantaricin A, a bacteriocin from *Lactobacillus plantarum* C11, is located on the same transcription unit as an agr-like regulatory system. *Appl. Environ. Microbiol.* 60 160–166. 8117074 10.1128/aem.60.1.160-166.1994 PMC201284

[B16] Fiocco D. Capozzi V. Collins M. Gallone A. Hols P. Guzzo J. (2010). Characterization of the CtsR stress response regulon in *Lactobacillus plantarum*. *J. Bacteriol.* 192 896–900. 10.1128/JB.01122-09 19933364 PMC2812460

[B17] Fiocco D. Collins M. Muscariello L. Hols P. Kleerebezem M. Msadek T. (2009). The *Lactobacillus plantarum* ftsH gene is a novel member of the CtsR stress response regulon. *J. Bacteriol.* 191 1688–1694. 10.1128/JB.01551-08 19074391 PMC2648225

[B18] Firth N. Fink P. D. Johnson L. Skurray R. A. (1994). A lipoprotein signal peptide encoded by the staphylococcal conjugative plasmid pSK41 exhibits an activity resembling that of Enterococcus faecalis pheromone cAD1. *J. Bacteriol.* 176 5871–5873. 10.1128/jb.176.18.5871-5873.1994 8083183 PMC196797

[B19] Fujii T. Ingham C. Nakayama J. Beerthuyzen M. Kunuki R. Molenaar D. (2008). Two homologous Agr-like quorum-sensing systems cooperatively control adherence, cell morphology, and cell viability properties in *Lactobacillus plantarum* WCFS1. *J. Bacteriol.* 190 7655–7665. 10.1128/JB.01489-07 18805979 PMC2583610

[B20] Gao Y. Duan J. Geng X. Zhang Z. Zhang R. Li X. (2017). Deficiency of quorum sensing system inhibits the resistance selection of *Pseudomonas aeruginosa* to ciprofloxacin and levofloxacin in vitro. *J. Glob. Antimicrob. Resist.* 10 113–119. 10.1016/j.jgar.2017.04.008 28729210

[B21] Goodson M. S. Harbaugh S. V. Chushak Y. G. Kelley-Loughnane N. (2015). Integrating and amplifying signal from riboswitch biosensors. *Methods Enzymol.* 550 73–91. 10.1016/bs.mie.2014.10.032 25605381

[B22] Gray K. M. Passador L. Iglewski B. H. Greenberg E. P. (1994). Interchangeability and specificity of components from the quorum-sensing regulatory systems of *Vibrio fischeri* and *Pseudomonas aeruginosa*. *J. Bacteriol.* 176 3076–3080. 10.1128/jb.176.10.3076-3080.1994 8188610 PMC205467

[B23] Gury J. Seraut H. Tran N. P. Barthelmebs L. Weidmann S. Gervais P. (2009). Inactivation of PadR, the repressor of the phenolic acid stress response, by molecular interaction with Usp1, a universal stress protein from *Lactobacillus plantarum*, in *Escherichia coli*. *Appl. Environ. Microbiol.* 75 5273–5283. 10.1128/AEM.00774-09 19542339 PMC2725474

[B24] Haddaji N. Mahdhi A. K. Ismaiil M. B. Bakhrouf A. (2017). Effect of environmental stress on cell surface and membrane fatty acids of *Lactobacillus plantarum*. *Arch. Microbiol.* 199 1243–1250. 10.1007/s00203-017-1395-9 28597197

[B25] Halleran A. D. Murray R. M. (2018). Cell-free and in vivo characterization of Lux, Las, and Rpa quorum activation systems in *E. coli*. *ACS Synth. Biol.* 7 752–755. 10.1021/acssynbio.7b00376 29120612

[B26] Hammer B. K. Bassler B. L. (2003). Quorum sensing controls biofilm formation in *Vibrio cholerae*. *Mol. Microbiol.* 50 101–104. 10.1046/j.1365-2958.2003.03688.x 14507367

[B27] Heunis T. Deane S. Smit S. Dicks L. M. (2014). Proteomic profiling of the acid stress response in *Lactobacillus plantarum* 423. *J. Proteome Res.* 13 4028–4039. 10.1021/pr500353x 25068841

[B28] Hunt S. M. Werner E. M. Huang B. Hamilton M. A. Stewart P. S. (2004). Hypothesis for the role of nutrient starvation in biofilm detachment. *Appl. Environ. Microbiol.* 70 7418–7425. 10.1128/AEM.70.12.7418-7425.2004 15574944 PMC535154

[B29] Ishihama Y. Oda Y. Tabata T. Sato T. Nagasu T. Rappsilber J. (2005). Exponentially modified protein abundance index (emPAI) for estimation of absolute protein amount in proteomics by the number of sequenced peptides per protein. *Mol. Cell Proteomics* 4 1265–1272. 10.1074/mcp.M500061-MCP200 15958392

[B30] Jahoor A. Patel R. Bryan A. Do C. Krier J. Watters C. (2008). Peroxisome proliferator-activated receptors mediate host cell proinflammatory responses to *Pseudomonas aeruginosa* autoinducer. *J. Bacteriol.* 190 4408–4415. 10.1128/JB.01444-07 18178738 PMC2446782

[B31] Janzon L. Arvidson S. (1990). The role of the delta-lysin gene (hld) in the regulation of virulence genes by the accessory gene regulator (agr) in *Staphylococcus aureus*. *EMBO J.* 9 1391–1399. 10.1002/j.1460-2075.1990.tb08254.x 2328718 PMC551825

[B32] Jensen R. O. Winzer K. Clarke S. R. Chan W. C. Williams P. (2008). Differential recognition of Staphylococcus aureus quorum-sensing signals depends on both extracellular loops 1 and 2 of the transmembrane sensor AgrC. *J. Mol. Biol.* 381 300–309. 10.1016/j.jmb.2008.06.018 18582472

[B33] Jensen V. Lons D. Zaoui C. Bredenbruch F. Meissner A. Dieterich G. (2006). RhlR expression in *Pseudomonas aeruginosa* is modulated by the *Pseudomonas* quinolone signal via PhoB-dependent and -independent pathways. *J. Bacteriol.* 188 8601–8606. 10.1128/JB.01378-06 17028277 PMC1698233

[B34] Jia F. F. Zheng H. Q. Sun S. R. Pang X. H. Liang Y. Shang J. C. (2018). Role of luxS in Stress Tolerance and Adhesion Ability in *Lactobacillus plantarum* KLDS1.*0391*. *Biomed. Res. Int.* 2018:4506829. 10.1155/2018/4506829 29651434 PMC5832066

[B35] Kanehisa M. Goto S. (2000). KEGG: kyoto encyclopedia of genes and genomes. *Nucleic Acids Res.* 28 27–30. 10.1093/nar/28.1.27 10592173 PMC102409

[B36] Kariminik A. Baseri-Salehi M. Kheirkhah B. (2017). *Pseudomonas aeruginosa* quorum sensing modulates immune responses: an updated review article. *Immunol. Lett.* 190 1–6. 10.1016/j.imlet.2017.07.002 28698104

[B37] Kleerebezem M. Quadri L. E. Kuipers O. P. De Vos W. M. (1997). Quorum sensing by peptide pheromones and two-component signal-transduction systems in Gram-positive bacteria. *Mol. Microbiol.* 24 895–904. 10.1046/j.1365-2958.1997.4251782.x 9219998

[B38] Ko J. S. Yang H. R. Chang J. Y. Seo J. K. (2007). *Lactobacillus plantarum* inhibits epithelial barrier dysfunction and interleukin-8 secretion induced by tumor necrosis factor-alpha. *World J. Gastroenterol.* 13 1962–1965. 10.3748/wjg.v13.i13.1962 17461497 PMC4146973

[B39] Krueger F. (2015). *Trim Galore: A Wrapper Tool Around Cutadapt and FastQC to Consistently Apply Quality and Adapter Trimming to FastQ Files*. Available at: http://www.bioinformatics.babraham.ac.uk/projects/trim_galore/ (accessed January 12, 2017).

[B40] Latifi A. Foglino M. Tanaka K. Williams P. Lazdunski A. (1996). A hierarchical quorum-sensing cascade in *Pseudomonas aeruginosa* links the transcriptional activators LasR and RhIR (VsmR) to expression of the stationary-phase sigma factor RpoS. *Mol. Microbiol.* 21 1137–1146. 10.1046/j.1365-2958.1996.00063.x 8898383

[B41] Lee K. Rho B. S. Pi K. Kim H. J. Choi Y. J. (2011). Proteomic analysis of protein expression in *Lactobacillus plantarum* in response to alkaline stress. *J. Biotechnol.* 153 1–7. 10.1016/j.jbiotec.2011.02.008 21356255

[B42] Lex A. Gehlenborg N. Strobelt H. Vuillemot R. Pfister H. (2014). UpSet: visualization of intersecting sets. *IEEE Trans. Vis. Comput. Graph.* 20 1983–1992. 10.1109/TVCG.2014.2346248 26356912 PMC4720993

[B43] Li L. Tetu S. G. Paulsen I. T. Hassan K. A. (2018). “A transcriptomic approach to identify novel drug efflux pumps in bacteria,” in *Bacterial Multidrug Exporters* eds Yamaguchi A. Nishino K. (New York, NY: Humana Press) 221–235. 10.1007/978-1-4939-7454-2_12 29177833

[B44] Love M. I. Huber W. Anders S. (2014). Moderated estimation of fold change and dispersion for RNA-seq data with DESeq2. *Genome Biol.* 15:550. 10.1186/s13059-014-0550-8 25516281 PMC4302049

[B45] Lubkowicz D. Ho C. L. Hwang I. Y. Yew W. S. Lee Y. S. Chang M. W. (2018). Reprogramming probiotic *Lactobacillus reuteri* as a biosensor for *Staphylococcus aureus* derived AIP-I detection. *ACS Synth. Biol.* 7 1229–1237. 10.1021/acssynbio.8b00063 29652493

[B46] Magoc T. Wood D. Salzberg S. L. (2013). EDGE-pro: estimated degree of gene expression in prokaryotic genomes. *Evol. Bioinform. Online* 9 127–136. 10.4137/EBO.S11250 23531787 PMC3603529

[B47] Maldonado A. Jimenez-Diaz R. Ruiz-Barba J. L. (2004a). Induction of plantaricin production in *Lactobacillus plantarum* NC8 after coculture with specific gram-positive bacteria is mediated by an autoinduction mechanism. *J. Bacteriol.* 186 1556–1564. 14973042 10.1128/JB.186.5.1556-1564.2004 PMC344433

[B48] Maldonado A. Ruiz-Barba J. L. Jimenez-Diaz R. (2004b). Production of plantaricin NC8 by *Lactobacillus plantarum* NC8 is induced in the presence of different types of gram-positive bacteria. *Arch. Microbiol.* 181 8–16. 14647979 10.1007/s00203-003-0606-8

[B49] Maldonado A. Ruiz-Barba J. L. Floriano B. Jimenez-Diaz R. (2002). The locus responsible for production of plantaricin S, a class IIb bacteriocin produced by *Lactobacillus plantarum* LPCO10, is widely distributed among wild-type Lact. plantarum strains isolated from olive fermentations. *Int. J. Food Microbiol.* 77 117–124. 10.1016/S0168-1605(02)00049-1 12076029

[B50] Maldonado A. Ruiz-Barba J. L. Jimenez-Diaz R. (2003). Purification and genetic characterization of plantaricin NC8, a novel coculture-inducible two-peptide bacteriocin from *Lactobacillus plantarum* NC8. *Appl. Environ. Microbiol.* 69 383–389. 10.1128/AEM.69.1.383-389.2003 12514019 PMC152457

[B51] Marchand N. Collins C. H. (2013). Peptide-based communication system enables *Escherichia coli* to Bacillus megaterium interspecies signaling. *Biotechnol. Bioeng.* 110 3003–3012. 10.1002/bit.24975 23775238

[B52] Marchand N. Collins C. H. (2016). Synthetic quorum sensing and cell-cell communication in gram-positive *Bacillus megaterium*. *ACS Synth. Biol.* 5 597–606. 10.1021/acssynbio.5b00099 26203497

[B53] Mathesius U. Mulders S. Gao M. Teplitski M. Caetano-Anolles G. Rolfe B. G. (2003). Extensive and specific responses of a eukaryote to bacterial quorum-sensing signals. *Proc. Natl. Acad. Sci. U.S.A.* 100 1444–1449. 10.1073/pnas.262672599 12511600 PMC298792

[B54] Matsumoto T. Tateda K. Miyazaki S. Furuya N. Ohno A. Ishii Y. (1997). Immunomodulating effect of fosfomycin on gut-derived sepsis caused by *Pseudomonas aeruginosa* in mice. *Antimicrob. Agents Chemother.* 41 308–313. 10.1128/AAC.41.2.308 9021184 PMC163706

[B55] Medina G. Juarez K. Diaz R. Soberon-Chavez G. (2003a). Transcriptional regulation of *Pseudomonas aeruginosa* rhlR, encoding a quorum-sensing regulatory protein. *Microbiology* 149 3073–3081. 10.1099/mic.0.26282-0 14600219

[B56] Medina G. Juarez K. Valderrama B. Soberon-Chavez G. (2003b). Mechanism of *Pseudomonas aeruginosa* RhlR transcriptional regulation of the rhlAB promoter. *J. Bacteriol.* 185 5976–5983. 10.1128/JB.185.20.5976-5983.2003 14526008 PMC225020

[B57] Miller M. B. Bassler B. L. (2001). Quorum sensing in bacteria. *Annu. Rev. Microbiol.* 55 165–199. 10.1146/annurev.micro.55.1.165 11544353

[B58] Nesvizhskii A. I. Keller A. Kolker E. Aebersold R. (2003). A statistical model for identifying proteins by tandem mass spectrometry. *Anal. Chem.* 75 4646–4658. 10.1021/ac0341261 14632076

[B59] Nguyen Y. N. Sheng H. Dakarapu R. Falck J. R. Hovde C. J. Sperandio V. (2013). The acyl-homoserine lactone synthase YenI from Yersinia enterocolitica modulates virulence gene expression in enterohemorrhagic *Escherichia coli* O157:H7. *Infect. Immun.* 81 4192–4199. 10.1128/IAI.00889-13 23980115 PMC3811827

[B60] Papakyriacou H. Vaz D. Simor A. Louie M. Mcgavin M. J. (2000). Molecular analysis of the accessory gene regulator (agr) locus and balance of virulence factor expression in epidemic methicillin-resistant Staphylococcus aureus. *J. Infect. Dis.* 181 990–1000. 10.1086/315342 10720522

[B61] Peng H. L. Novick R. P. Kreiswirth B. Kornblum J. Schlievert P. (1988). Cloning, characterization, and sequencing of an accessory gene regulator (agr) in Staphylococcus aureus. *J. Bacteriol.* 170 4365–4372. 10.1128/jb.170.9.4365-4372.1988 2457579 PMC211451

[B62] Peral M. C. Martinez M. A. Valdez J. C. (2009). Bacteriotherapy with *Lactobacillus plantarum* in burns. *Int. Wound J.* 6 73–81. 10.1111/j.1742-481X.2008.00577.x 19291120 PMC7951207

[B63] Peral M. C. Rachid M. M. Gobbato N. M. Huaman Martinez M. A. Valdez J. C. (2010). Interleukin-8 production by polymorphonuclear leukocytes from patients with chronic infected leg ulcers treated with *Lactobacillus plantarum*. *Clin. Microbiol. Infect.* 16 281–286. 10.1111/j.1469-0691.2009.02793.x 19519855

[B64] Pereira C. S. Thompson J. A. Xavier K. B. (2013). AI-2-mediated signalling in bacteria. *FEMS Microbiol. Rev.* 37 156–181. 10.1111/j.1574-6976.2012.00345.x 22712853

[B65] Perkins D. N. Pappin D. J. Creasy D. M. Cottrell J. S. (1999). Probability-based protein identification by searching sequence databases using mass spectrometry data. *Electrophoresis* 20 3551–3567. 10.1002/(SICI)1522-2683(19991201)20:18<3551::AID-ELPS3551>3.0.CO;2-2 10612281

[B66] Pessi G. Haas D. (2000). Transcriptional control of the hydrogen cyanide biosynthetic genes hcnABC by the anaerobic regulator ANR and the quorum-sensing regulators LasR and RhlR in *Pseudomonas aeruginosa*. *J. Bacteriol.* 182 6940–6949. 10.1128/JB.182.24.6940-6949.2000 11092854 PMC94819

[B67] Pieterse B. Leer R. J. Schuren F. H. Van Der Werf M. J. (2005). Unravelling the multiple effects of lactic acid stress on *Lactobacillus plantarum* by transcription profiling. *Microbiology* 151 3881–3894. 10.1099/mic.0.28304-0 16339934

[B68] Procaccini A. Lunt B. Szurmant H. Hwa T. Weigt M. (2011). Dissecting the specificity of protein-protein interaction in bacterial two-component signaling: orphans and crosstalks. *PLoS One* 6:e19729. 10.1371/journal.pone.0019729 21573011 PMC3090404

[B69] Puertollano E. Puertollano M. A. Cruz-Chamorro L. Alvarez De Cienfuegos G. Ruiz-Bravo A. De Pablo M. A. (2008). Orally administered *Lactobacillus plantarum* reduces pro-inflammatory interleukin secretion in sera from Listeria monocytogenes infected mice. *Br. J. Nutr.* 99 819–825. 10.1017/S0007114507832533 17894920

[B70] Ramos A. N. Cabral M. E. Noseda D. Bosch A. Yantorno O. M. Valdez J. C. (2012). Antipathogenic properties of *Lactobacillus plantarum* on *Pseudomonas aeruginosa*: the potential use of its supernatants in the treatment of infected chronic wounds. *Wound Repair Regen.* 20 552–562. 10.1111/j.1524-475X.2012.00798.x 22642376

[B71] Ramos A. N. Gobbato N. Rachid M. Gonzalez L. Yantorno O. Valdez J. C. (2010). Effect of *Lactobacillus plantarum* and *Pseudomonas aeruginosa* culture supernatants on polymorphonuclear damage and inflammatory response. *Int. Immunopharmacol.* 10 247–251. 10.1016/j.intimp.2009.11.007 19932196

[B72] Ramos A. N. Sesto Cabral M. E. Arena M. E. Arrighi C. F. Arroyo Aguilar A. A. Valdez J. C. (2015). Compounds from *Lactobacillus plantarum* culture supernatants with potential pro-healing and anti-pathogenic properties in skin chronic wounds. *Pharm. Biol.* 53 350–358. 10.3109/13880209.2014.920037 25347359

[B73] Rampioni G. Bertani I. Zennaro E. Polticelli F. Venturi V. Leoni L. (2006). The quorum-sensing negative regulator RsaL of *Pseudomonas aeruginosa* binds to the lasI promoter. *J. Bacteriol.* 188 815–819. 10.1128/JB.188.2.815-819.2006 16385073 PMC1347304

[B74] Rampioni G. Schuster M. Greenberg E. P. Bertani I. Grasso M. Venturi V. (2007). RsaL provides quorum sensing homeostasis and functions as a global regulator of gene expression in *Pseudomonas aeruginosa*. *Mol. Microbiol.* 66 1557–1565. 10.1111/j.1365-2958.2007.06029.x 18045385

[B75] Rampioni G. Schuster M. Greenberg E. P. Zennaro E. Leoni L. (2009). Contribution of the RsaL global regulator to *Pseudomonas aeruginosa* virulence and biofilm formation. *FEMS Microbiol. Lett.* 301 210–217. 10.1111/j.1574-6968.2009.01817.x 19878323

[B76] Reading N. C. Sperandio V. (2006). Quorum sensing: the many languages of bacteria. *FEMS Microbiol. Lett.* 254 1–11. 10.1111/j.1574-6968.2005.00001.x 16451172

[B77] Rekhif N. Atrih A. Lefebvre G. (1995). Activity of plantaricin SA6, a bacteriocin produced by *Lactobacillus plantarum* SA6 isolated from fermented sausage. *J. Appl. Bacteriol.* 78 349–358. 10.1111/j.1365-2672.1995.tb03417.x 7744719

[B78] Reyes D. Andrey D. O. Monod A. Kelley W. L. Zhang G. Cheung A. L. (2011). Coordinated regulation by AgrA, SarA, and SarR to control agr expression in Staphylococcus aureus. *J. Bacteriol.* 193 6020–6031. 10.1128/JB.05436-11 21908676 PMC3194896

[B79] Ritchie A. J. Jansson A. Stallberg J. Nilsson P. Lysaght P. Cooley M. A. (2005). The *Pseudomonas aeruginosa* quorum-sensing molecule N-3-(oxododecanoyl)-L-homoserine lactone inhibits T-cell differentiation and cytokine production by a mechanism involving an early step in T-cell activation. *Infect. Immun.* 73 1648–1655. 10.1128/IAI.73.3.1648-1655.2005 15731065 PMC1064928

[B80] Ritchie A. J. Whittall C. Lazenby J. J. Chhabra S. R. Pritchard D. I. Cooley M. A. (2007). The immunomodulatory *Pseudomonas aeruginosa* signalling molecule N-(3-oxododecanoyl)-L-homoserine lactone enters mammalian cells in an unregulated fashion. *Immunol. Cell Biol.* 85 596–602. 10.1038/sj.icb.7100090 17607318

[B81] Ritchie A. J. Yam A. O. Tanabe K. M. Rice S. A. Cooley M. A. (2003). Modification of in vivo and in vitro T- and B-cell-mediated immune responses by the *Pseudomonas aeruginosa* quorum-sensing molecule N-(3-oxododecanoyl)-L-homoserine lactone. *Infect. Immun.* 71 4421–4431. 10.1128/IAI.71.8.4421-4431.2003 12874321 PMC165988

[B82] Rumbaugh K. P. Griswold J. A. Hamood A. N. (1999a). Contribution of the regulatory gene lasR to the pathogenesis of *Pseudomonas aeruginosa* infection of burned mice. *J. Burn. Care Rehabil.* 20 42–49. 9934636 10.1097/00004630-199901001-00008

[B83] Rumbaugh K. P. Griswold J. A. Iglewski B. H. Hamood A. N. (1999b). Contribution of quorum sensing to the virulence of *Pseudomonas aeruginosa* in burn wound infections. *Infect. Immun.* 67 5854–5862. 10531240 10.1128/iai.67.11.5854-5862.1999 PMC96966

[B84] Schauder S. Shokat K. Surette M. G. Bassler B. L. (2001). The LuxS family of bacterial autoinducers: biosynthesis of a novel quorum-sensing signal molecule. *Mol. Microbiol.* 41 463–476. 10.1046/j.1365-2958.2001.02532.x 11489131

[B85] Schultz M. Veltkamp C. Dieleman L. A. Grenther W. B. Wyrick P. B. Tonkonogy S. L. (2002). *Lactobacillus plantarum* 299V in the treatment and prevention of spontaneous colitis in interleukin-10-deficient mice. *Inflamm. Bowel Dis.* 8 71–80. 10.1097/00054725-200203000-00001 11854603

[B86] Searle B. C. (2010). Scaffold: a bioinformatic tool for validating MS/MS-based proteomic studies. *Proteomics* 10 1265–1269. 10.1002/pmic.200900437 20077414

[B87] Seed P. C. Passador L. Iglewski B. H. (1995). Activation of the *Pseudomonas aeruginosa* lasI gene by LasR and the *Pseudomonas* autoinducer PAI: an autoinduction regulatory hierarchy. *J. Bacteriol.* 177 654–659. 10.1128/jb.177.3.654-659.1995 7836299 PMC176640

[B88] Seme H. Gjuracic K. Kos B. Fujs S. Stempelj M. Petkovic H. (2015). Acid resistance and response to pH-induced stress in two *Lactobacillus plantarum* strains with probiotic potential. *Benef. Microbes* 6 369–379. 10.3920/BM2014.0069 25380802

[B89] Semmelhack M. F. Campagna S. R. Hwa C. Federle M. J. Bassler B. L. (2004). Boron binding with the quorum sensing signal AI-2 and analogues. *Org. Lett.* 6 2635–2637. 10.1021/ol048976u 15255709

[B90] Serrano L. M. Molenaar D. Wels M. Teusink B. Bron P. A. De Vos W. M. (2007). Thioredoxin reductase is a key factor in the oxidative stress response of *Lactobacillus plantarum* WCFS1. *Microb. Cell Fact* 6:29. 10.1186/1475-2859-6-29 17725816 PMC2174512

[B91] Sharif D. I. Gallon J. Smith C. J. Dudley E. (2008). Quorum sensing in Cyanobacteria: N-octanoyl-homoserine lactone release and response, by the epilithic colonial cyanobacterium Gloeothece PCC6909. *ISME J.* 2 1171–1182. 10.1038/ismej.2008.68 18633449

[B92] Smith R. S. Harris S. G. Phipps R. Iglewski B. (2002a). The *Pseudomonas aeruginosa* quorum-sensing molecule N-(3-oxododecanoyl)homoserine lactone contributes to virulence and induces inflammation in vivo. *J. Bacteriol.* 184 1132–1139. 11807074 10.1128/jb.184.4.1132-1139.2002 PMC134808

[B93] Smith R. S. Kelly R. Iglewski B. H. Phipps R. P. (2002b). The *Pseudomonas* autoinducer N-(3-oxododecanoyl) homoserine lactone induces cyclooxygenase-2 and prostaglandin E2 production in human lung fibroblasts: implications for inflammation. *J. Immunol.* 169 2636–2642. 12193735 10.4049/jimmunol.169.5.2636

[B94] Storey D. G. Ujack E. E. Rabin H. R. Mitchell I. (1998). *Pseudomonas aeruginosa* lasR transcription correlates with the transcription of lasA, lasB, and toxA in chronic lung infections associated with cystic fibrosis. *Infect. Immun.* 66 2521–2528. 9596711 10.1128/iai.66.6.2521-2528.1998 PMC108233

[B95] Sturme M. H. Francke C. Siezen R. J. De Vos W. M. Kleerebezem M. (2007). Making sense of quorum sensing in lactobacilli: a special focus on *Lactobacillus plantarum* WCFS1. *Microbiology* 153 3939–3947. 10.1099/mic.0.2007/012831-0 18048908

[B96] Sturme M. H. Nakayama J. Molenaar D. Murakami Y. Kunugi R. Fujii T. (2005). An agr-like two-component regulatory system in *Lactobacillus plantarum* is involved in production of a novel cyclic peptide and regulation of adherence. *J. Bacteriol.* 187 5224–5235. 10.1128/JB.187.15.5224-5235.2005 16030216 PMC1196011

[B97] Taga M. E. Semmelhack J. L. Bassler B. L. (2001). The LuxS-dependent autoinducer AI-2 controls the expression of an ABC transporter that functions in AI-2 uptake in *Salmonella typhimurium*. *Mol. Microbiol.* 42 777–793. 10.1046/j.1365-2958.2001.02669.x 11722742

[B98] Teplitski M. Mathesius U. Rumbaugh K. P. (2011). Perception and degradation of N-acyl homoserine lactone quorum sensing signals by mammalian and plant cells. *Chem. Rev.* 111 100–116. 10.1021/cr100045m 20536120

[B99] Tong Y. Zhai Q. Lu W. Tian F. Zhao J. Zhang H. (2017). New insights in integrated response mechanism of *Lactobacillus plantarum* under excessive manganese stress. *Food Res. Int.* 102 323–332. 10.1016/j.foodres.2017.10.014 29195955

[B100] Valdez J. C. Peral M. C. Rachid M. Santana M. Perdigon G. (2005). Interference of *Lactobacillus plantarum* with *Pseudomonas aeruginosa* in vitro and in infected burns: the potential use of probiotics in wound treatment. *Clin. Microbiol. Infect.* 11 472–479. 10.1111/j.1469-0691.2005.01142.x 15882197

[B101] Van Bokhorst-van de Veen H. Bongers R. S. Wels M. Bron P. A. Kleerebezem M. (2013). Transcriptome signatures of class I and III stress response deregulation in *Lactobacillus plantarum* reveal pleiotropic adaptation. *Microb. Cell Fact* 12:112. 10.1186/1475-2859-12-112 24238744 PMC3842655

[B102] Vendeville A. Winzer K. Heurlier K. Tang C. M. Hardie K. R. (2005). Making ’sense’ of metabolism: autoinducer-2, LuxS and pathogenic bacteria. *Nat. Rev. Microbiol.* 3 383–396. 10.1038/nrmicro1146 15864263

[B103] Wagner C. Zimmermann S. Brenner-Weiss G. Hug F. Prior B. Obst U. (2007). The quorum-sensing molecule N-3-oxododecanoyl homoserine lactone (3OC12-HSL) enhances the host defence by activating human polymorphonuclear neutrophils (PMN). *Anal. Bioanal. Chem.* 387 481–487. 10.1007/s00216-006-0698-5 16906383

[B104] Waters C. M. Bassler B. L. (2005). Quorum sensing: cell-to-cell communication in bacteria. *Annu. Rev. Cell Dev. Biol.* 21 319–346. 10.1146/annurev.cellbio.21.012704.131001 16212498

[B105] Waters C. M. Bassler B. L. (2006). The Vibrio harveyi quorum-sensing system uses shared regulatory components to discriminate between multiple autoinducers. *Genes Dev.* 20 2754–2767. 10.1101/gad.1466506 17015436 PMC1578700

[B106] Wen K. Y. Cameron L. Chappell J. Jensen K. Bell D. J. Kelwick R. (2017). A Cell-Free Biosensor for Detecting Quorum Sensing Molecules in *P. aeruginosa*-Infected Respiratory Samples. *ACS Synth. Biol.* 6 2293–2301. 10.1021/acssynbio.7b00219 28981256

[B107] Wickham H. (2016). *ggplot2: Elegant Graphics for Data Analysis*. Berlin: Springer 10.1007/978-3-319-24277-4

[B108] Winzer K. Falconer C. Garber N. C. Diggle S. P. Camara M. Williams P. (2000). The *Pseudomonas aeruginosa* lectins PA-IL and PA-IIL are controlled by quorum sensing and by RpoS. *J. Bacteriol.* 182 6401–6411. 10.1128/JB.182.22.6401-6411.2000 11053384 PMC94786

[B109] Winzer K. Williams P. (2001). Quorum sensing and the regulation of virulence gene expression in pathogenic bacteria. *Int. J. Med. Microbiol.* 291 131–143. 10.1078/1438-4221-00110 11437336

[B110] Xia Y. Chen H. Q. Zhang M. Jiang Y. Q. Hang X. M. Qin H. L. (2011). Effect of *Lactobacillus plantarum* LP-Onlly on gut flora and colitis in interleukin-10 knockout mice. *J. Gastroenterol. Hepatol.* 26 405–411. 10.1111/j.1440-1746.2010.06498.x 21261733

[B111] Yates E. A. Philipp B. Buckley C. Atkinson S. Chhabra S. R. Sockett R. E. (2002). N-acylhomoserine lactones undergo lactonolysis in a pH-, temperature-, and acyl chain length-dependent manner during growth of Yersinia pseudotuberculosis and *Pseudomonas aeruginosa*. *Infect. Immun.* 70 5635–5646. 10.1128/IAI.70.10.5635-5646.2002 12228292 PMC128322

[B112] Zhang G. Zhang F. Ding G. Li J. Guo X. Zhu J. (2012). Acyl homoserine lactone-based quorum sensing in a methanogenic archaeon. *ISME J.* 6 1336–1344. 10.1038/ismej.2011.203 22237544 PMC3379639

[B113] Zhu J. Miller M. B. Vance R. E. Dziejman M. Bassler B. L. Mekalanos J. J. (2002). Quorum-sensing regulators control virulence gene expression in *Vibrio cholerae*. *Proc. Natl. Acad. Sci. U.S.A.* 99 3129–3134. 10.1073/pnas.052694299 11854465 PMC122484

